# Rational Constraints and the Evolution of Fairness in the Ultimatum Game

**DOI:** 10.1371/journal.pone.0134636

**Published:** 2015-07-30

**Authors:** Damon Tomlin

**Affiliations:** Department of Psychology, University of Colorado Colorado Springs, Colorado Springs, Colorado, United States of America; Universidad Carlos III de Madrid, SPAIN

## Abstract

Behavior in the Ultimatum Game has been well-studied experimentally, and provides a marked contrast between the theoretical model of a self-interested economic agent and that of an actual human concerned with social norms such as fairness. How did such norms evolve, when punishing unfair behavior can be costly to the punishing agent? The work described here simulated a series of Ultimatum Games, in which populations of agents earned resources based on their preferences for proposing and accepting (or rejecting) offers of various sizes. Two different systems governing the acceptance or rejection of offers were implemented. Under one system, the probability that an agent accepted an offer of a given size was independent of the probabilities of accepting the other possible offers. Under the other system, a simple, ordinal constraint was placed on the acceptance probabilities such that a given offer was at least as likely to be accepted as a smaller offer. For simulations under either system, agents’ preferences and their corresponding behavior evolved over multiple generations. Populations without the ordinal constraint came to emulate maximizing economic agents, while populations with the constraint came to resemble the behavior of human players.

## Introduction

The field of behavioral economics has allowed the scientific community to study the robustness of many social norms [1], as well as the factors which influence their enforcement [2–4]. One of the most heavily employed tasks in behavioral economics is the Ultimatum Game (UG) [5], a simple decision-making task involving two people that pits economic self-interest against social norms of fairness. In this task, one agent, the “proposer,” has a resource that he or she must split with another agent, the “responder.” The proposer offers a fraction of the resource, and the responder either accepts or rejects the offer. If it is accepted, the two agents receive the proposed fractions of the resource; if it is rejected, both agents receive nothing. An economically “rational” responder should accept any non-zero offer, since something is invariably better than nothing, and proposers should therefore offer the smallest non-zero amount.

This policy differs markedly from observed human behavior in this task, as human players routinely reject low offers [6]. The apparent impact of norms for fairness in economic exchanges [7] has prompted a great deal of work demonstrating that such norms exist both across species [8] and across human cultures, although these norms have been shown to vary from one culture to another in the context of the UG [9–11]. This pattern of behavior has also given rise to another question: does this behavior reflect emotionally-driven impulses that have evolved to reinforce equitable distributions of resources (regardless of the potential cost of such reinforcement), or is it a more consciously strategic decision-making process that seeks to maximize payoffs?

Bodies of research exist in support of both points. Empirical data and mathematical models that place an inherent value on equity have been used to lend support to the idea that humans place a premium on fairness in and of itself [12–16]. Furthermore, behavioral experiments have been conducted demonstrating that when a group member violates these norms, other members will take measures to punish the offending individual, even when doing so incurs a non-trivial cost to the punisher [17]. However, research also suggests that players’ behavior can be brought more in line with that predicted for the economically rational agent by changing the information available to the player, or by allowing the player to adjust his or her behavior over the course of multiple rounds of the game [18–21]. Recent work has even begun to elucidate the mechanisms within the human brain that produce and/or influence these behaviors [22–24]. Regardless of the specific level of cognition necessary for adherence to these norms, how did evolutionary processes allow this behavior, in which decision-makers opt to forego valuable resources, to succeed?

Previous research has used computational approaches to address this question. It has been shown that rejection of unfair offers and the prevalence of fair ones in a simulated environment can be achieved variously by using an augmented utility function that rewards rejections [25], setting spatial constraints that create sub-communities within the population [26], allowing agents the capacity for empathy [27], granting agents information regarding a partner’s past behavior [28] or implementing a two-stage learning process so that agents can test strategies offline [29]. However, could norms regarding fairness evolve in groups of agents without placing them under exogenous constraints or granting them additional cognitive abilities?

The work presented here implements such computational agents and tests the impact of a simple, rational constraint on agents’ acceptance rates: that agents should be at least as open to a given offer as they would be to a lower one. That is, what is the impact of acceptance rates that increase monotonically as a function of offer size? Prior computational studies of the UG have tended to define agents as having a single value that they can propose and a minimum acceptable offer (MAO) that they will accept [28–30]. Behavioral studies themselves vary as to whether they query the participant for such an MAO, or allow the participant to respond to offers as they are made. Defining an agent by its MAO does impose monotonicity on its acceptance rates, but this monotonicity comes in a very specific, inflexible form: a step function for which the acceptance rate is 0% below the MAO, and 100% at or above it. Does this particular functional form reflect the evolutionary equilibrium at which complex agents would arrive?

Other computational studies of the UG have examined the role of noise in agents’ behavior. Prior work by Gale et al. [29] and more recent work by Rand et al. [31] have highlighted the potential importance of stochasticity in the evolution of norms for fairness. Their results demonstrate that the amount of noise (whether it is represented as perceptual noise within an agent or a mutation rate within a population) can have a measurable impact upon agents’ behavior. However, both of these studies employed the aforementioned MAO framework, leaving one to wonder whether the amount of noise in the system is as important when proposed offers, and their corresponding acceptance rates, take on a wider variety of functional forms.

Perhaps the answers to some of the above questions can be determined (or at least suggested) by building upon these models: that is, through combining stochastic mechanisms with the capacity for a broad range of behaviors by the agent, rather than embedding agents in an exotic environment where agents themselves are deterministically governed by one or two parameters. The work presented here compares the effects of two types of functional forms for acceptance rates: those that are constrained to be monotonically increasing (without the further imposition of an MAO-like heuristic), and those that are not.

## Results

### Evolution of populations with non-monotonic acceptance rates

**Fig 1:**
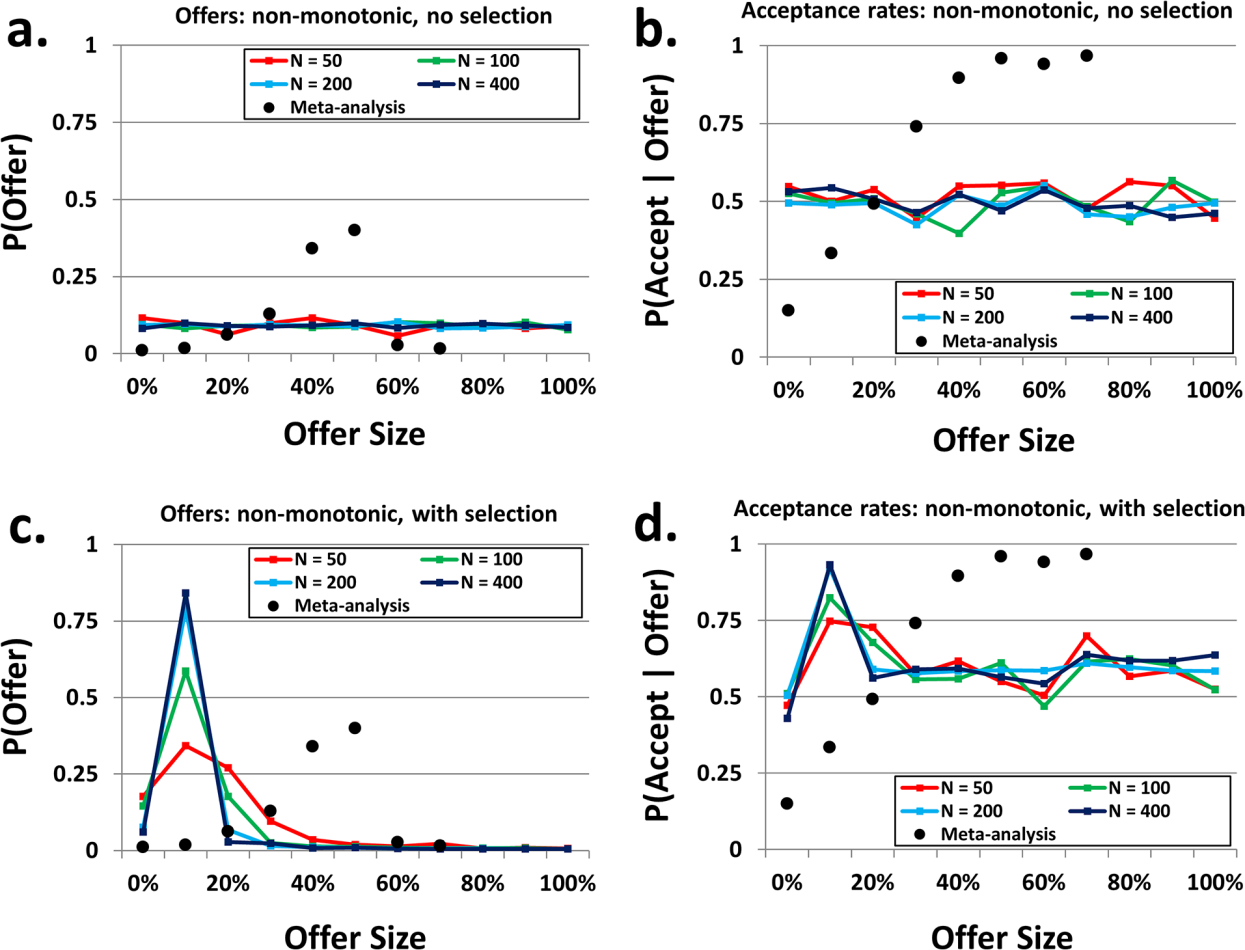
Final genotypes for non-monotonic acceptance rates. The lines in each panel show the mean genotype for each population size across the eleven possible offers. For each panel, black dots denote offer frequencies and conditional acceptance rates of human subjects, according to a meta-analysis of multiple behavioral studies. Because offers greater than 65% are rare in human studies, the rightmost dot comprises all offers of 65% or greater. **(a)** Frequencies of proposed offers without selection pressure. When the identities of reproducing agents were determined randomly, offer sizes were all equally frequent. **(b)** Rates of acceptance, conditional upon offer size, without selection pressure. When the identities of reproducing agents were determined randomly, acceptance rates averaged approximately 50% for all offer sizes. **(c)** Frequencies of proposed offers with selection pressure. When the probability of reproduction was proportional to earnings, populations evolved such that low offers dominated, as expected for maximizing agents. Larger populations adhered to low offers more strictly than smaller populations. **(d)** Rates of acceptance, conditional upon offer size, with selection pressure. When the probability of reproduction was proportional to earnings, acceptance rates increased for all non-zero offers, with the highest acceptance rates corresponding to the commonly proposed, low offers.

Fig 1 depicts the mean genotypes across the eleven possible offer sizes, after 500,000 generations, of populations of “non-monotonic” agents: those whose acceptance rates in the UG were independent of one another. As described in the Methods section, both the non-monotonic and monotonic simulations had an accompanying control, in which reproduction of the agents was determined without selection pressure (i.e., chosen at random with no regard for the earnings of each agent in the generation). Panel 1a demonstrates that, under this control condition, agents’ offers did not vary appreciably across the possible offer sizes. Similarly, panel 1b demonstrates that the absence of selection pressure resulted in acceptance rates of approximately 50% (conditional upon the offer size) for all offers, with no consistent pattern across offer sizes or population sizes. However, this lack of variation changed when selection pressure was introduced, as shown in panels 1c and 1d.

When populations reproduced in accordance with their earnings, genotypes governing behavior in the proposer role were heavily skewed toward low offers (panel 1c). Additionally, the lowest possible non-zero offer, that of 10 percent, was increasingly favored as population size was increased. A meta-analysis based upon ten existing studies of the Ultimatum Game calculated the mean frequencies of proposed offers by actual human players (see the Methods section for details regarding this analysis). The mean frequencies of proposed offers by actual human players are plotted (panel 1c, black dots) alongside the mean offer frequencies of the simulated agents. These data demonstrate that the preponderance of low offers yielded by the simulation, while predicted for economically rational agents, does not capture the behavior of actual human proposers. Panel 1d depicts the acceptance rates corresponding to these offers. Although there was not a large amount of variation across the possible offers, the acceptance rates were higher than those that evolved without selection pressure (excepting offers of zero), especially those corresponding to low, but frequent, offers. Mean acceptance rates for actual humans, as determined by the same meta-analysis, are also plotted (panel 1d, black dots). As in panel 1c, these data are not particularly well captured by the genotypes of simulated populations.

### Evolution of populations with monotonic acceptance rates

**Fig 2:**
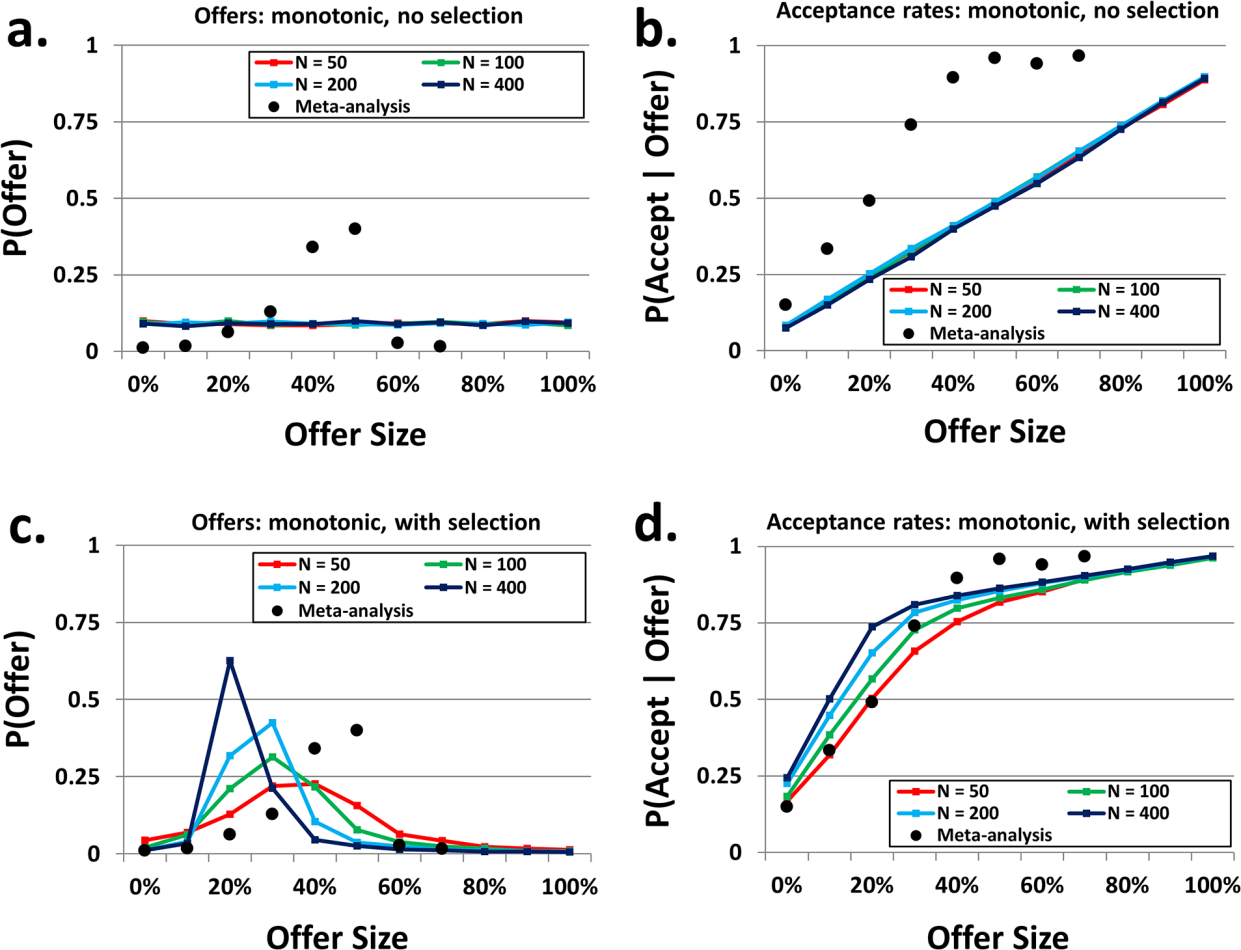
Final genotypes for monotonic acceptance rates. The lines in each panel show the mean genotype for each population size across the eleven possible offers. For each panel, black dots denote offer frequencies and conditional acceptance rates of human subjects, according to a meta-analysis of multiple behavioral studies. Because offers greater than 65% are rare in human studies, the rightmost dot comprises all offers of 65% or greater. **(a)** Frequencies of proposed offers without selection pressure. When the identities of reproducing agents were determined randomly, offer sizes were all equally frequent. **(b)** Rates of acceptance, conditional upon offer size, without selection pressure. When the identities of reproducing agents were determined randomly, but acceptance rates were constrained to be monotonically increasing, acceptance rates became linear with respect to offer size. **(c)** Frequencies of proposed offers with selection pressure. In contrast to the non-monotonic simulations, behavior shifts toward fairer offers, with modal offer sizes of 30%-40%. As in the non-monotonic simulations, larger populations tended toward lower offers than smaller populations. **(d)** Rates of acceptance, conditional upon offer size, with selection pressure. When the probability of reproduction was proportional to earnings, acceptance rates became non-linear with offer size, with concavity increasing with population size.

Fig 2 depicts the mean genotypes, after 500,000 generations, of populations of “monotonic” agents: those whose acceptance rates in the UG were constrained such that the acceptance rate for a given offer was at least as high as that for a lower offer. Panel a demonstrates that, as with the non-monotonic agents, when no selection pressure existed agents’ offers did not vary significantly across the possible offer sizes. Because of the monotonic constraint, however, panel 2b differs markedly from its counterpart in Fig 1. Here, mean acceptance rates across agents increased linearly across offer sizes, ranging from seven percent for offers of zero up to an acceptance rate of approximately 90 percent for offers of the entire resource. This linear pattern existed because the acceptance rates were randomly generated subject to the monotonic constraint, and thanks to the large number of simulations each increment in the offer size corresponds to an average increase in acceptance probability of 1 / (number of possible offers + 1), or approximately .08.

When these populations reproduced in accordance with their earnings, genotypes governing behavior in the proposer role exhibited significant variation (panel 2c). Here, the fairness of the offers improved dramatically over those in the non-monotonic simulations, with sizes of 30 or 40 percent being the modal offer. As with the non-monotonic populations, proposers in larger populations tended to make slightly smaller offers. Mean frequencies of proposed offers by actual human players (as determined by the same meta-analysis as above) are plotted along with these data (panel 2c, black dots), and the frequencies of offers yielded by the simulations are a much better fit to the human data than those produced by the non-monotonic simulations (although they are admittedly not a perfect fit; see the discussion section for more on this discrepancy). Panel 2d depicts the acceptance rates corresponding to these offers. The curvature of the lines, particularly noticeable in large populations, is a clear departure from the acceptance functions of panel 2b, which follow a simple, linear pattern. Here, the offers of 50 percent or more were accepted with high probability (80 percent or more) even in small populations, whose responders were more demanding.

It is also clear from panel 2d that the mean acceptance rates are not well represented by a step function that a single MAO would produce, even though such a step function is possible in the monotonic simulations (and the non-monotonic ones, for that matter). While this does not necessarily indicate that the mean was not comprised of a distribution of such MAO’s, in these simulations the individual genotypes were similar to the mean and were not well represented by step functions on either an agent-by-agent or population-by-population basis, either (see S1 and S2 Figs for a sample of the genotypes from individual agents and populations, respectively). Mean acceptance rates for actual humans, as determined by the above meta-analysis, are again plotted (panel 2d, black dots). The acceptance rates described by the mean genotype, especially those produced by smaller populations, provide a much better match to the real world data.

### Co-evolution of norms across time

**Fig 3:**
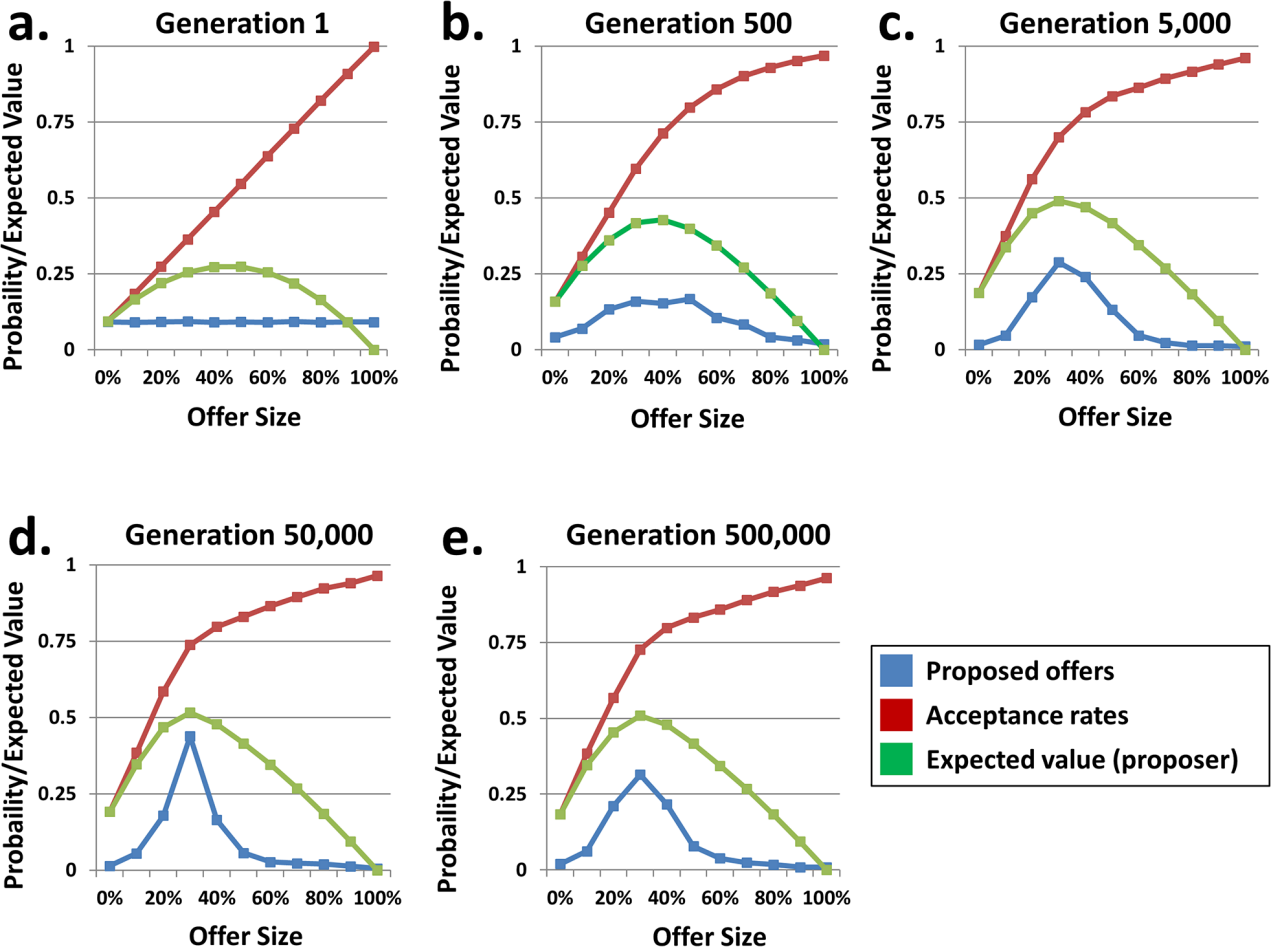
Co-evolution of offer frequencies and acceptance rates in monotonic populations with selection pressure. The lines in each panel show the mean genotype for proposers (blue line) and responders (red line) across the eleven possible offers for a population of size N = 100. The expected value of each offer, from the proposer’s perspective, is shown in green. When acceptance rates were constrained to monotonically increase over offer sizes, the highest expected value for proposers came from fair (or nearly fair) offers. As proposers and responders co-evolved, extremely high and extremely low offers ceased and proposers settled on a modal offer of 30%—the offer with the highest expected value.

As shown above, the imposition of monotonicity on acceptance rates not only produced acceptance rates similar to actual human behavior, but also shifted the modal proposed offer from the minimum non-zero offer of 10% (Fig 1C) to more fair offers of 30% (Fig 2C). However, the data above do not demonstrate *how* populations achieved this endpoint. Fig 3 depicts the evolutionary time course of proposer and responder genotypes for a single population size (N = 100). When the population was initialized, mean offers did not differ significantly across the possible offer sizes, and acceptance rates increased linearly across sizes due to the constraint of monotonicity (Fig 3A). From the proposers’ perspective, extremely low and high offers both had low expected values: the former because the likelihood of acceptance was very low, and the latter because the return to the proposer was very low. In contrast, the expected value of fairer offers was maximal. Proposers quickly evolved to avoid low and high offers in favor of fair ones (Fig 3B and 3C). Meanwhile, selection strongly favored responders that accepted fair or hyperfair offers. The probability of acceptance for low offers drifted upward as well, but there was no strong selection for this class of offers–they were not frequently proposed, and even when accepted they did not contribute much to the responders’ overall fitness (Fig 3B and 3C). The expected value for low offers from the proposer’s perspective continued to sharpen, and the genotypes for proposed offers followed suit. Upon reaching equilibrium (Fig 3E), offers of 30% accounted for more than half of all offers, with a negligible number of minimum non-zero offers being made (for evidence that the simulations indeed attained equilibrium after 500,000 generations, see S3 Fig).

The evolutionary trajectory described above did not, however, hold for populations with non-monotonic acceptance rates. Without an ordinal constraint, the acceptance rates across offer sizes drifted independently and the highest expected value, from the proposer’s perspective, came from proposing the lowest non-zero offer: 10% (see S4 Fig). Even when the non-monotonic populations were initialized with monotonically increasing acceptance rates (using the same procedure as those employed for monotonic populations), agents still evolved toward a modal offer of 10% (see S5 Fig). Additional simulations revealed that different initial conditions did not appreciably affect the final outcome of evolution for monotonic populations: populations evolved fairer modal offers even when they initially accepted no offers, all offers, or all non-zero offers (see S6 Fig). Furthermore, modal offers of 30% were attained by monotonic populations even when both proposer *and* responder genotypes were initialized to the rational policy of proposing and accepting the minimum non-zero offer (10%; see S7 Fig).

It is worth noting that evolving a high frequency of fairer offers depended critically on the co-evolution of proposers and responders. If the genotypes of proposers or responders were not allowed to evolve (e.g., proposers were constrained to make low offers, or responders were constrained to accept only hyperfair offers), the genotype of the other role indeed conformed to the rigid behavior (see S8 Fig). As noted above, however, as long as the behavior of both roles was allowed to evolve, the initial conditions governing responder genotypes did not have a noticeable impact upon the final norms.

Finally, the development of these norms did not rely upon each individual having an equal opportunity to occupy the proposer and responder roles (and therefore did not depend critically on the agents’ diversifying their earnings across the two roles). That is, it could be suggested that the majority of earnings were derived from agents’ choices as the proposer, and that consequently behavior evolved to maximize proposer fitness at the expense of the responder. Agents would have been insensitive to such a tradeoff in the evolutionary sense because they occupied both roles equally and therefore were not affected by a disparity in earnings. In order to test this idea, simulations were run in which agents occupied (and derived fitness from) only one of the roles, and thus would not have been insensitive to such a disparity. Even when agents were so constrained, agent behavior evolved toward the same modal offer of 30% (see S9 Fig). For an additional summary of the earnings (i.e., fitness) derived by each role in each of the conditions from Figs 1 and 2, see S10 Fig.

## Discussion

In the work presented here, populations of simulated agents interacted via the UG, earned resources in accordance with their performance and reproduced over the course of generations. The behavior of each agent was governed by its “genotype:” in this case, a series of values that described its likelihood of offering, and accepting, each of eleven possible splits of a resource. At the end of this evolutionary process, their genotypes (and the behavior represented by those genotypes) were analyzed. Agents whose acceptance rates were completely independent of one another evolved an approximation of economically rational behavior: low offers were commonly proposed and accepted. If these acceptance rates were subjected to a small constraint, such that a given offer was at least as likely to be accepted as a lower offer, then offers proposed during the interactions were significantly fairer. Analysis of the evolutionary trajectories of agents show that this constraint altered the distribution of acceptance rates and the corresponding expected values of the offers available to proposers, thus causing the two roles to co-evolve an equilibrium of fairer offers. Additional simulations revealed that different initial conditions did not appreciably affect these outcomes (see S5, S6 and S7 Figs). It is also worth noting that, for populations with monotonically increasing acceptance rates, the functional forms of the offers proposed and the frequency of acceptance by the responders capture several important features found in laboratory studies of humans playing the UG.

The results do not match the human data perfectly, however, especially when it comes to the distribution of proposed offers. While the modal offer was 30 percent for the simulated agents, the modal offer in human studies is 40 to 50 percent. One proposed reason for such high offers in economic studies is the effect of the experimental observer [32, 33]—a third party that was absent in the simulated interactions here–although the effect of such an observer in the UG is a matter of some debate [34]. Another omission from the computational environment was that of culture, which has been shown to have measurable effect on fairness-related behavior [9–11]. Finally, the manner in which the task is framed has also been reported to have a significant impact on offer size [35], but no manipulations of framing were tested in these simulations.

Many prior studies (such as Gale et al., Iranzo et al., Nowak et al. and Page et al. [26–29]) have employed a simple model of responder behavior in which acceptance or rejection of an offer was determined by a single parameter: the minimum acceptable offer, or MAO. Such an operationalization is powerful thanks to its simplicity (which allows the application of methods such as the replicator equation, which could not be used here). However, is this level of description sufficient to capture human behavior? Recapitulating human behavior under this framework seems to necessitate the addition of environmental or cognitive constraints: a two-phase learning system with perceptual noise (Gale et al.; 29), a capacity for empathy (Iranzo et al.; 27), an ability to access information regarding other agents’ previous behavior (Nowak et al.; 28), or a spatial constraint that formed small sub-communities (Page et al.; 26). In contrast, by withholding or imposing a simple constraint endogenous to the game (i.e., one that governed choice behavior within the UG itself, rather than exerting its influence in domains not inherent to the UG), agents exhibited behavior that was similar either to economically rational agents or to actual human participants, respectively. Although the monotonically constrained agents evolved acceptance rates similar to those exhibited by human decision-makers, the functional forms of neither the mean across the population nor the individual agents provided a good match to an MAO model of responder behavior.

Previous research, most notably by Gale et al. [29] and Rand et al. [31], has indicated the impact of stochasticity on agents’ behavior under the MAO framework. As shown in S11 Fig, the amount of noise in the simulations presented here (represented as a mutation rate) did not alter the agents’ modal offer or the functional form of their acceptance rates at steady state. This finding further demonstrates that, while MAO models are advantageous in their simplicity, they may also produce phenomena that do not generalize to agents not constrained by such a framework. Results such as these are, perhaps, an advantage of using agents that not only have access to a wide space of decisions but can exhibit a larger array of behaviors within that space.

In addition to emphasizing the difference between agents under the MAO framework and those with a larger array of options, the findings presented here may also explain a difference between the social behavior of humans and that of other species. It has been previously reported that chimpanzees, while closely related to humans, nevertheless behave as rational economic agents in the UG [36]. If such a difference exists, then the explanation must lie in an environmental or cognitive factor specific to humans. Factors such as perceptual noise (as studied by Gale et al. [29]) and spatial proximity (as studied by Page et al. [26]), while they have been shown to be influential, would come into play for a variety of species. Explicit, ordinal preferences, such as those studied here, require a degree of sophisticated cognition that is more likely to be restricted to humans (capacities such as empathy [27] are another potential source of the difference between humans and other species).

It is worth pointing out that even the behaviorally rich agents presented above were subject to several assumptions. First, the method demonstrated here treated each member of the population as equally likely to be in the role of proposer or responder. What if the members within a group were not so homogenous, but rather tended toward one role or the other? If such an imbalance existed, how would the heritability of the roles themselves affect reciprocity and norms for fairness? Determining the impact of such imbalances between the roles is an avenue for future study.

A second assumption in the model presented here was that the agents had no memory of their prior interactions. Previous work has highlighted the importance of reputation as it pertains to the UG, and shown that information on players’ past behavior can have a significant effect upon norms of fairness [28]. How would these effects be altered by the imposition of monotonic acceptance rates? Would knowledge of agents’ past behavior have to be publicly broadcast for it to have an effect, or could private knowledge between interacting agents suffice to enforce fairness? In the simulations presented here, fitness was determined by the expected value of exchanges and was therefore insensitive to the number of interactions within a generation. In order to investigate the role of publicly versus privately held knowledge, new simulations would need to consider the amount of experience possessed by agents by enumerating individual interactions, as well as the means by which reputational information is transmitted. On a more speculative note, what if behavioral or morphological traits that are not obviously relevant can actually yield clues as to the agent’s likely behavior, and how does the reliability of these cues affect agents’ decisions?

The answers to these questions are not obvious, nor is it obvious which constraints can be placed safely on the cognition of simulated agents in the Ultimatum Game. As the answers are revealed, however, they may inform our understanding of how our now-prevalent social norms came to be, and how these norms will continue to evolve in the future.

## Methods

### Agent genotypes

For the first generation of a simulated population, agents were initialized with a random “genotype” comprised of 22 values drawn from a uniform distribution ranging from zero to one. Eleven of these values determined the probability that the agent, when acting as proposer, made an offer of each possible size: from zero to 100 percent in ten percent increments. This probability distribution summed to one over the eleven values. The other eleven values in the genotype determined the probability that the agent would accept each of the possible offers (conditional on the offer being made), and ranged from probabilities of zero to one. With regard to these acceptance rates, there were two different classes of simulation. In “non-monotonic” simulations, each of the agent’s eleven acceptance rates was independent of the others. In “monotonic” simulations, acceptance rates were constrained to be *monotonically increasing* as a function of offer size. That is, for two offers of different sizes, the acceptance rate for the larger offer was at least as high as that for the smaller offer. It is important to note that this constraint neither forced low acceptance rates for low offers, nor forced high acceptance rates for offers of any size. Under this system, acceptance rates across offer sizes could take on many functional forms, including a “step function” equivalent to an MAO heuristic.

### Computation of agent earnings

Calculation of the agents’ earnings (which, as described below, were synonymous with fitness) was performed via a simple calculation of expected value. This method assumed that each agent was equally likely to be in the proposer role as in the responder role, that each agent was equally likely to interact with every other agent and that the amount of resource being split was one unit. For each agent, the mean acceptance rates and offer probabilities were computed over the entire population (excluding the agent itself, as agents were not allowed to interact with themselves) according to each agent’s genotype. The total expected earnings for agent *j* as proposer were:

$$Earnings_{proposer\;j}=\sum_{i=0}^{10}\left(1-\frac{i}{10}\right)*{\bar{A}}_{i}*P_{ij}$$ (1)

where ${\bar{A}}_{i}$ is the population’s mean acceptance rate (excluding agent *j*) for offer *i* and *P*_*ij*_ is agent *j*’s probability of proposing offer *i*. Similarly, the total expected earnings for agent *j* as responder were:

$$Earnings_{responder\;j}=\sum_{i=0}^{10}\frac{i}{10}*A_{ij}*{\bar{P}}_{i}$$ (2)

where *A*_*ij*_ is agent *j*’s probability of accepting offer *i* and ${\bar{P}}_{i}$ is the population’s mean probability of proposing offer *i* (excluding agent *j*). The two sources of earnings (proposer and responder) were equally weighted according to the assumption above. Note that this method did not require that the number of rounds played by each agent be specified–only that the numbers of rounds played by the agents were equal, on average.

### Initialization of the populations

Agents were initialized randomly with respect to their behavior in the role of proposer: the eleven loci governing the offers they proposed were assigned values generated from a uniform distribution of random numbers and normalized such that they summed to one. For their behavior in the role of responder, the initial acceptance rates were generated according to the constraints of the relevant simulation. For non-monotonic populations, each of the eleven loci that governed acceptance of each offer size was assigned a value generated from a uniform distribution of random numbers ranging from zero to one. For populations whose acceptance rates were monotonically constrained, the eleven loci were similarly assigned random values. After this step, however, those agents whose eleven values summed to greater than one were normalized such that the cumulative probability (i.e., the probability of accepting an offer of 100%) was equal to one. Although monotonic populations could not be initialized with non-monotonic acceptance functions, additional simulations were implemented in which non-monotonic populations were initialized with monotonically increasing acceptance functions (using the same procedure described above; see S5 Fig). Monotonic populations were also simulated after initialization with three alternative acceptance functions: one under which no offers were accepted, one under which all offers were accepted and one under which all non-zero offers were accepted (i.e., an economically rational policy; see S6 Fig).

### Reproduction and evolution of the population

Populations of agents reproduced according to a Wright-Fisher process [37]. For simulations in which selection pressure was based on earnings, the probability of a given agent producing offspring for the next generation was equal to the fraction represented by the agent’s own earnings, relative to the population’s total earnings. As a control condition, simulations were also run without selection pressure. For these simulations, every agent had an equal probability of reproducing. The number of offspring created by each agent was determined stochastically according to these probabilities, with the number of agents in the population remaining constant over the generations.

In addition, each offspring carried a probability of .2 of mutating from its parent’s genotype (lower mutation rates of .01 and .1 and a higher mutation rate of .4 were also tested, but did not appreciably affect the results; see S11 Fig). If an offspring was a mutant, one of the 22 loci in the agent’s genotype was selected at random to change. If the selected locus governed proposer behavior, the selected locus was altered by .05 (randomly selected to be positive or negative) and the probability distribution was renormalized (resulting in a total difference of .1 between the offspring’s and parent’s genotype); because monotonic and non-monotonic did not differ in terms of proposer behavior, the same procedure was used for both. If the selected locus governed responder behavior and the population’s acceptance rates were non-monotonic, the selected locus was altered by .1; because values at the eleven loci governing acceptance rates were independent, this similarly resulted in a total difference of .1 between the offspring’s and parent’s genotype. If the selected locus governed responder behavior and the population’s acceptance rates were constrained to be monotonically increasing, the selected locus was altered by .05 and the probability distribution was renormalized if the probability of accepting an offer of 100% was greater than one (again, resulting in a total difference of .1 between the offspring’s and parent’s genotype). Because acceptance rates in these populations were constrained to be monotonically increasing, any change in a given locus was carried over to loci corresponding to higher offers. Loci with new values less than zero or greater than one were readjusted to these bounds. Using this schema, populations reproduced and mutated over the course of 500,000 generations. For each data point presented here, 1,000 different populations were simulated.

### Analysis of results

Once the last generation was simulated, the genotypes and earnings of the last generation of agents were averaged. These means were themselves averaged over the 1,000 different populations simulated under each population size and simulation type (the 2 x 2 combination of [non-monotonic, monotonic] and [without selection pressure, with selection pressure]). These means consequently correspond to the average offer frequency, and acceptance rates, across the populations at the end of the evolutionary process. For an examination of the final genotypes of individual populations (rather than the combined means of 1,000 different populations), see S1 Fig; for an examination of the final genotypes of individual agents, see S2 Fig.

### Meta-analysis of previous research

In order to compare the results of these simulations to actual human behavior in the UG, published data from ten different behavioral studies were used to construct average offer frequencies and acceptance rates [3, 5, 11, 32, 38–43]. The list of evaluated articles was based upon an existing review of the relevant literature [6], an online search of scholarly articles, and citations found in articles so identified. To be included in the meta-analysis, studies were required to contain data from actual human participants and to publish their data in such a form that that the frequency of proposed offers and their corresponding acceptance rates could be extracted from figures and/or tables (i.e., articles could not simply present the mean accepted offer). This requirement was implemented so that the human data could be directly compared to the genotypes of simulated agents, which specified offer frequencies and acceptance rates over a range of offer sizes.

In addition, the experimental methods of the studies had to meet four criteria. First, if the study employed a multi-round version of the Ultimatum Game, participants in each round had to be paired with a different partner (or at least believe that this was the case), and involvement in each round had to be independent of the outcome of previous rounds. This prerequisite allowed the meta-analysis to avoid biases stemming from reputation effects or participants’ knowledge of the conditions for entry into subsequent rounds. Second, proposers in each round of the study had to be able to choose from a large number of possible offers, including hyperfair offers; many implementations of the Ultimatum Game force the proposer to make a binary choice between an even split of the resource and a low offer. This requirement made the behavior of human proposers comparable to that of the simulated proposers presented here, which could make any of eleven different offers ranging from nothing to the entire resource. Third, responders in each study had to base decisions on a specific offer, rather than indicate responses to a range of hypothetical offers before being matched randomly with a proposer. The latter approach presents participants with a more complicated problem, as it encourages proposers to estimate the distribution of acceptance rates across the entire population. This constraint was implemented in order to yield a data set comparable to the simulated agents, whose behavior was not based on any meta-knowledge of the population. Finally, in order to be included in the meta-analysis, participants had to know the potential payoffs to both proposers and responders. This requirement allowed the comparison of the behavior of human responders to that of simulated responders, who experienced no uncertainty with regard to the amount earned by each player in the exchange. For articles that published the results of multiple experimental manipulations, only experiments whose manipulations conformed to the guidelines above were included.

After extracting the behavioral information from each article, data were combined at the level of individual decisions. Mean proposal frequencies and acceptance rates, binned in ten percent increments, were calculated from this comprehensive data set, mitigating the impact of smaller sample sizes that would occur if mean behavior was calculated for each study and then averaged over studies. In order to average over as many studies as possible, this meta-analysis ignored the role of nationality, culture, total amount of resource to be divided, and variations in the instructions given to participants. As such, estimated mean proposal frequencies and acceptance rates could be biased by these variables, and such bias would vary with the number of participants in each study.

## Supporting Information

S1 PRISMA Checklist PRISMA Checklist for Meta-analysis of Existing Ultimatum Game Studies. A detailed listing of procedures employed in the meta-analysis and where each is described in the article.
(PDF)

S1 Fig Genotypes of individual populations. The mean genotypes are shown for forty of the 1,000 populations that were simulated with N = 100 agents. For panels a, b, d and e, each line represents the mean genotype of an individual population. **(a)** Frequencies of proposed offers for non-monotonic populations with selection pressure. **(b)** Rates of acceptance, conditional upon offer size, for non-monotonic populations with selection pressure. **(c)** Distribution of modal offers for non-monotonic populations with selection pressure. Although there was some variability in the modal offers across the individual populations, modal offers of 10% (the lowest non-zero offer) were by far the most frequent. **(d)** Frequencies of proposed offers for monotonic populations with selection pressure. **(e)** Rates of acceptance, conditional upon offer size, for monotonic populations with selection pressure. As can be seen, acceptance rates for individual populations exhibited patterns similar to the overall mean; that is, the mean across the populations was not comprised of a collection of minimum acceptable offers (MAO’s). **(f)** Distribution of modal offers for monotonic populations with selection pressure. Although modal offers of 30% were the most frequent, modal offers of 20% and 40% were also common.
(TIF)

S2 Fig Genotypes of individual agents within a sample population. The mean genotypes are shown for forty of the 100 agents in a single population. For all panels, each line represents the mean genotype of an individual agent. **(a)** Frequencies of proposed offers for non-monotonic populations with selection pressure. **(b)** Rates of acceptance, conditional upon offer size, for non-monotonic populations with selection pressure. **(c)** Frequencies of proposed offers for monotonic populations with selection pressure. **(d)** Rates of acceptance, conditional upon offer size, for monotonic populations with selection pressure. As was the case for individual populations, acceptance rates for individual agents did not resemble a collection of minimum acceptable offers (MAO’s).
(TIF)

S3 Fig Time courses of genotypes over 500,000 generations. For all panels, each line represents the mean genotype across populations for each of the eleven offer sizes (N = 100 agents). Data are plotted across 500,000 generations of the simulation. As shown above, both non-monotonic and monotonic populations exhibited no systematic drift by the end of the simulations. **(a)** Mean genotypes for proposed offers in non-monotonic populations with selection pressure. **(b)** Mean genotypes for acceptance rates in non-monotonic populations with selection pressure. **(c)** Mean genotypes for proposed offers in monotonic populations with selection pressure. **(d)** Mean genotypes for acceptance rates in monotonic populations with selection pressure.
(TIF)

S4 Fig Co-evolution of offer proposals and acceptance rates in non-monotonic populations with selection pressure. The sequence of panels depicts the evolutionary time course of proposer and responder genotypes for a single population size (N = 100 agents) when responders’ acceptance rates were not constrained to increase monotonically across offer sizes. The lines in each panel show the mean genotype for proposers (blue line) and responders (red line) across the eleven possible offers. The expected value of each offer, from the proposer’s perspective, is shown in green. When the population was initialized, neither mean offers nor acceptance rates differed significantly across the possible magnitudes and the highest expected value for proposers initially came from an offer of zero. However, the maximum expected value quickly shifted to the minimum non-zero offer (10%), as responders derived no fitness from accepting offers of zero, but did derive a small amount of fitness from low offers. As proposers and responders co-evolved, offers of 10% accounted for approximately half of all offers, with a negligible number of offers over 30% being made.
(TIF)

S5 Fig Non-monotonic populations with monotonic initialization for acceptance rates. For each panel, mean genotypes of 1,000 populations (N = 100 agents) are shown for proposed offers (blue) and acceptance rates (red) after 500,000 generations. All responder genotypes were constrained to be monotonically increasing across offer sizes. **(a)** Data from the original, non-monotonic populations, with selection (Fig 1C and 1D), for purposes of comparison. **(b)** Data from simulations of non-monotonic populations that were initialized according to the procedure for monotonic populations. After initialization, populations evolved without the monotonic constraint on acceptance rates. After 500,000 generations, the resulting equilibria were not appreciably different: the most frequent offer was the lowest non-zero offer (i.e., 10% of the resource), and these low offers were frequently accepted.
(TIF)

S6 Fig Monotonic populations with alternate initializations for acceptance rates. Here, a variety of alternative conditions are tested to examine the robustness of the evolution of fair offers for monotonic populations. For each panel, mean genotypes of 1,000 populations (N = 100 agents) are shown for proposed offers (blue) and acceptance rates (red) after 500,000 generations. Genotypes for acceptance rates were constrained to be monotonically increasing across offer sizes. As shown above, none of these alternate initializations appreciably changed the final equilibrium: proposers still evolved a modal offer of 30% and the functional forms of acceptance rates were similar to the original initialization. **(a)** Data from the original, monotonically constrained simulations, with selection pressure (Fig 2C and 2D). **(b)** Data from simulations in which responders initially rejected all offers (i.e., acceptance rates were initialized to zero for all loci). **(c)** Data from simulations in which responders initially accepted all offers (i.e., acceptance rates were initialized to one for all loci). **(d)** Data from simulations in which responders initially accepted all non-zero offers (i.e., acceptance rates were initialized to zero for the locus corresponding to 0%, and one for all other loci).
(TIF)

S7 Fig Monotonic populations with economically rational initialization for both roles. Here, an additional alternative condition is tested, in which both responder and proposer genotypes were initialized to the economically rational equilibrium. Responders were initialized to accept all non-zero offers (i.e., acceptance rates were initialized to zero for the locus corresponding to 0%, and one for all other loci) and proposed offers were initialized such that offers of 10% had a probability of one. For each panel, mean genotypes of 1,000 populations (N = 100 agents) are shown for proposed offers (blue) and acceptance rates (red) after 500,000 generations. Acceptance rates were constrained to be monotonically increasing across offer sizes. **(a)** Data from the original, monotonically constrained simulations, with selection pressure (Fig 2C and 2D). **(b)** Data from simulations with the economically rational initialization. This alternate initialization did not appreciably change the final equilibrium: proposers still evolved a modal offer of 30% and the functional forms of acceptance rates were similar to the original initialization.
(TIF)

S8 Fig Monotonic populations without co-evolution. Here, alternative conditions are tested to examine the adaptability of population genotypes when only one role was allowed to evolve. For each panel, mean genotypes of 1,000 populations (N = 100 agents) are shown for proposed offers (blue) and acceptance rates (red) after 500,000 generations. Acceptance rates were constrained to be monotonically increasing across offer sizes. **(a)** Data from the original, monotonically constrained simulations (Fig 2C and 2D). **(b)** When proposers were constrained to make only low offers (with genotypes based on those from non-monotonic populations, Fig 1C) and did not evolve, acceptance rates for the minimum non-zero offer (10% of the resource) shifted from 38% to 48%. **(c)** When responders were initialized to accept only hyperfair offers and did not evolve, proposer genotypes evolved to make modal offers of 70%. Thus, each role could indeed adapt to the behavior of the other, and the genotypes exhibited by the original, monotonically constrained populations depended on the co-evolution of both roles.
(TIF)

S9 Fig Monotonic populations with separate, heritable roles. For each panel, mean genotypes of 1,000 populations (N = 100 agents) are shown for proposed offers (blue) and acceptance rates (red) after 500,000 generations. Acceptance rates were constrained to be monotonically increasing across offer sizes. **(a)** Data from the original, monotonically constrained simulations, with selection pressure (Fig 2C and 2D). **(b)** When proposers and responders were two separate but simultaneously evolving populations, results were similar (but not identical) to those in Fig 2C and 2D: the modal offer was 30%, and these offers were frequently accepted (although not as frequently as in the original simulation). That is, the norms co-evolved by the populations did not depend critically on the individuals’ ability to diversify the source of their fitness across the two roles.
(TIF)

S10 Fig Comparison of earnings across simulations and roles. Mean earnings for proposers and responders are shown, grouped by the type of simulation. The earnings of agents that did not experience any form of selection pressure did not vary when acceptance rates were non-monotonic. For non-monotonic populations with selection (i.e., when acceptance rates were not monotonically constrained and reproduction depended upon earnings), proposers earned significantly more than responders, with the disparity accentuated for larger populations. This is not surprising, given the prevalence of low offers and their high rates of acceptance–a norm to which agents more strictly adhere as the population size increases. Earnings did not vary according to population size in the monotonically constrained populations without selection pressure. However, responders did earn more than proposers, as offers were uniformly distributed and acceptance rates for high offers were mathematically constrained to be higher than those for low offers. For monotonic populations with selection (i.e., when acceptance rates were monotonically constrained and reproduction depended upon earnings), the disparity in earnings between the roles was mitigated relative to non-monotonic populations with selection.
(TIF)

S11 Fig Monotonic populations with alternate mutation rates. For panels a and d, mean genotypes of 1,000 populations (N = 100 agents) are shown for proposed offers (a) and acceptance rates (d) for multiple mutation rates. In addition to the nominal mutation rate used for all other simulations (a value of .2), mutation rates of .01, .1 and .4 (i.e., the inverse of the population size, half of the nominal rate, and double the nominal rate) were simulated with selection pressure. Acceptance rates were constrained to be monotonically increasing across offer sizes in all three simulations. For panels b, c, e and f, each line represents the mean genotype across populations for each of the eleven offer sizes (N = 100 agents). Data are plotted across 500,000 generations for the high mutation rate (panels c and f); in order to ensure that steady state was reached, simulations with the very low mutation rate were run for 1,000,000 generations and are plotted accordingly. **(a)** Mean offer probabilities under the four mutation rates. Modal offers remained at 30% regardless of the mutation rate possessed by the population. **(b)** Mean genotypes for proposed offers with a very low mutation rate. As shown above, offers exhibited no systematic drift by the end of the simulations. **(c)** Mean genotypes for proposed offers with a high mutation rate. As shown above, offers exhibited no systematic drift by the end of the simulations. **(d)** Mean acceptance rates under the four mutation rates. Although acceptance rates were slightly higher for lower mutation rates, the functional forms were qualitatively the same. **(e)** Mean genotypes for acceptance rates with a very low mutation rate. As shown above, acceptance rates exhibited no systematic drift by the end of the simulations. **(f)** Mean genotypes for acceptance rates with a high mutation rate. As shown above, acceptance rates exhibited no systematic drift by the end of the simulations.
(TIF)

S12 Fig PRISMA Flow Diagram for Meta-analysis of Existing Ultimatum Game Studies. Details regarding the screening of studies used to summarize human behavior in the Ultimatum Game.
(PDF)
