# Supplementary figures and images for: Rational Constraints and the Evolution of Fairness in the Ultimatum Game
Source: PLoS One. 2015 Jul 30;10(7):e0134636. doi: 10.1371/journal.pone.0134636 (PMC4520471; doi:10.1371/journal.pone.0134636)

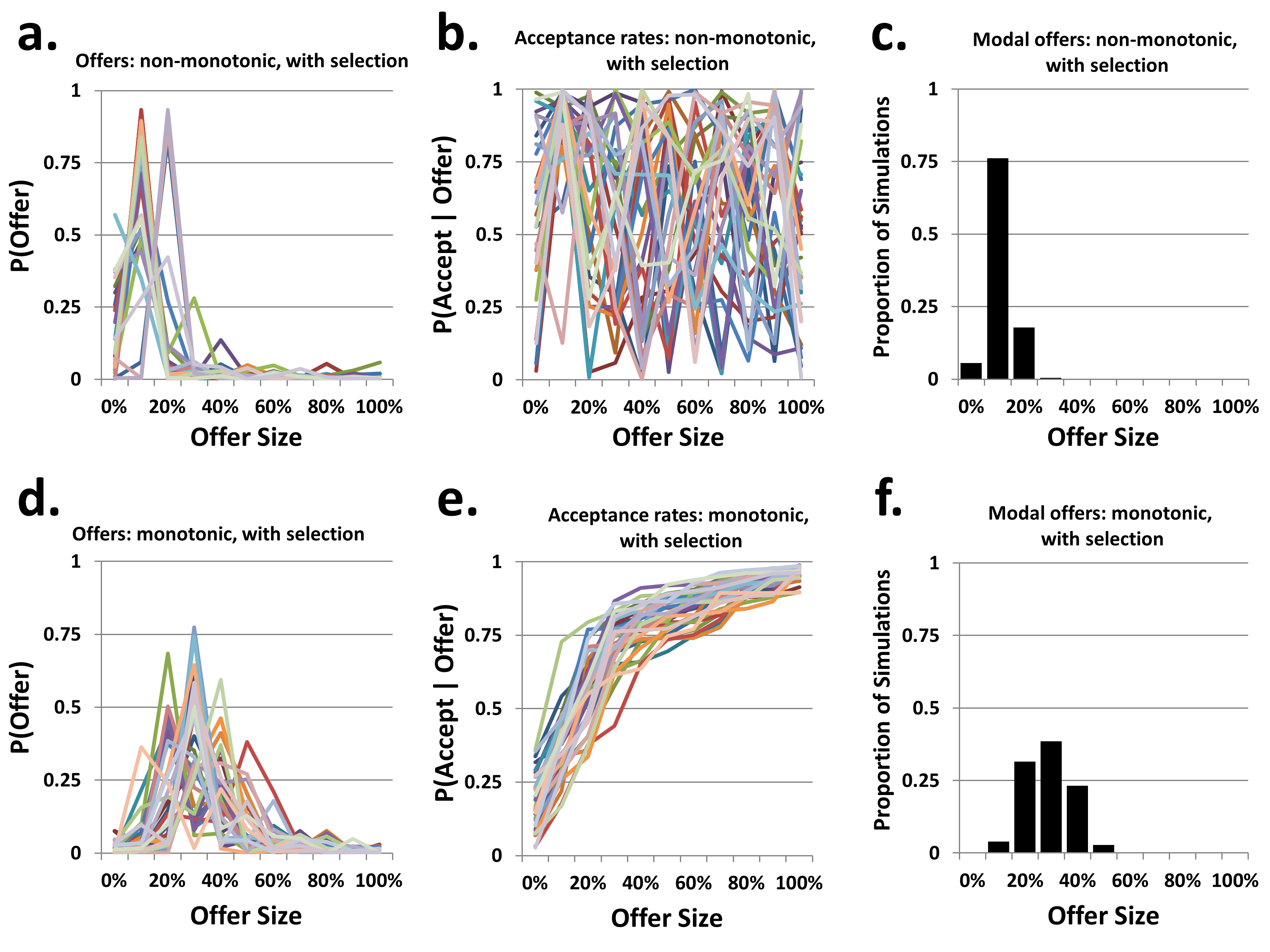

Supplement: S1 Fig — The mean genotypes are shown for forty of the 1,000 populations that were simulated with N = 100 agents. For panels a, b, d and e, each line represents the mean genotype of an individual population. (a) Frequencies of proposed offers for non-monotonic populations with selection pressure. (b) Rates of acceptance, conditional upon offer size, for non-monotonic populations with selection pressure. (c) Distribution of modal offers for non-monotonic populations with selection pressure. Although there was some variability in the modal offers across the individual populations, modal offers of 10% (the lowest non-zero offer) were by far the most frequent. (d) Frequencies of proposed offers for monotonic populations with selection pressure. (e) Rates of acceptance, conditional upon offer size, for monotonic populations with selection pressure. As can be seen, acceptance rates for individual populations exhibited patterns similar to the overall mean; that is, the mean across the populations was not comprised of a collection of minimum acceptable offers (MAO’s). (f) Distribution of modal offers for monotonic populations with selection pressure. Although modal offers of 30% were the most frequent, modal offers of 20% and 40% were also common. (TIF) [file pone.0134636.s002.TIF]

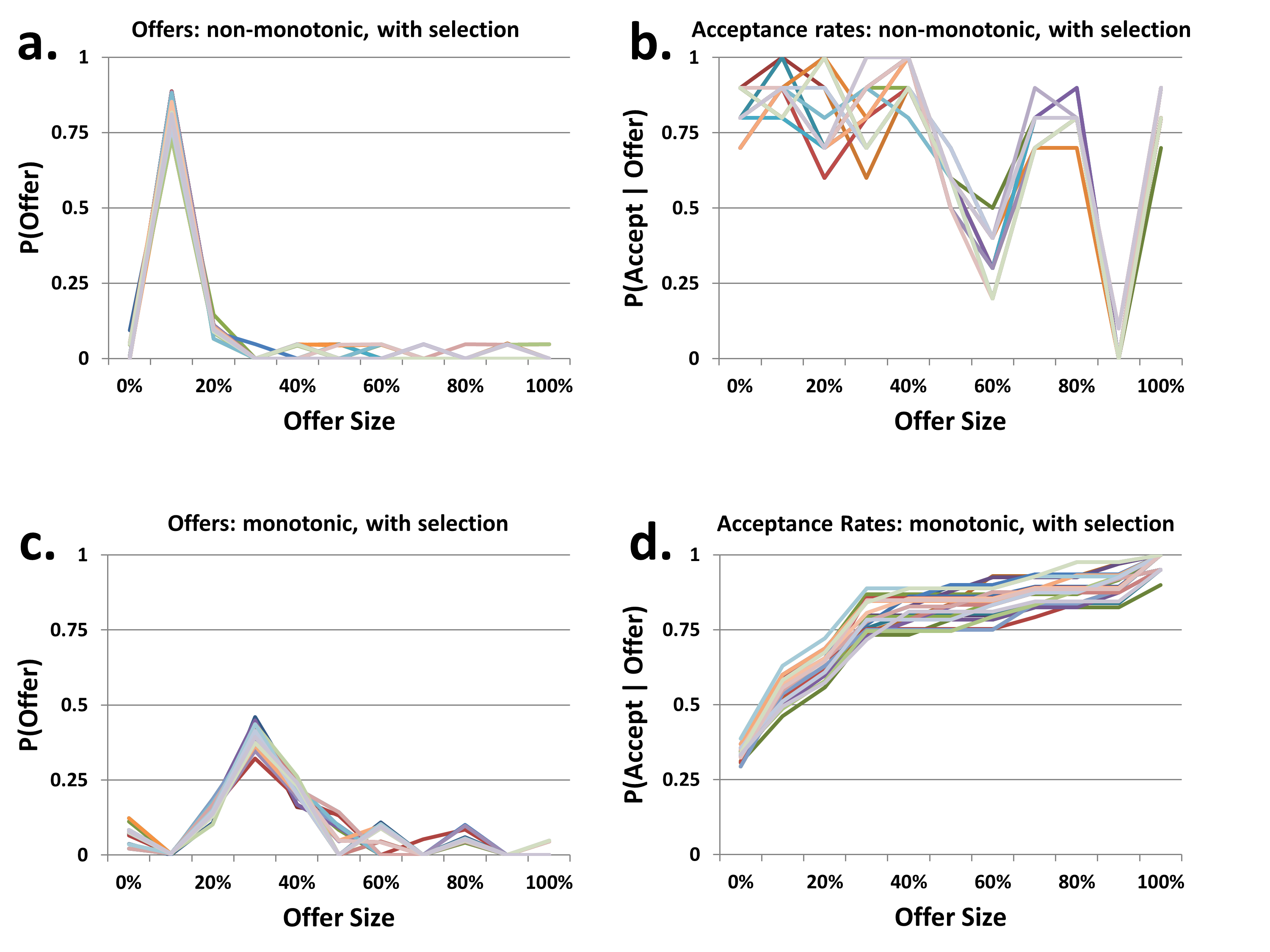

Supplement: S2 Fig — The mean genotypes are shown for forty of the 100 agents in a single population. For all panels, each line represents the mean genotype of an individual agent. (a) Frequencies of proposed offers for non-monotonic populations with selection pressure. (b) Rates of acceptance, conditional upon offer size, for non-monotonic populations with selection pressure. (c) Frequencies of proposed offers for monotonic populations with selection pressure. (d) Rates of acceptance, conditional upon offer size, for monotonic populations with selection pressure. As was the case for individual populations, acceptance rates for individual agents did not resemble a collection of minimum acceptable offers (MAO’s). (TIF) [file pone.0134636.s003.TIF]

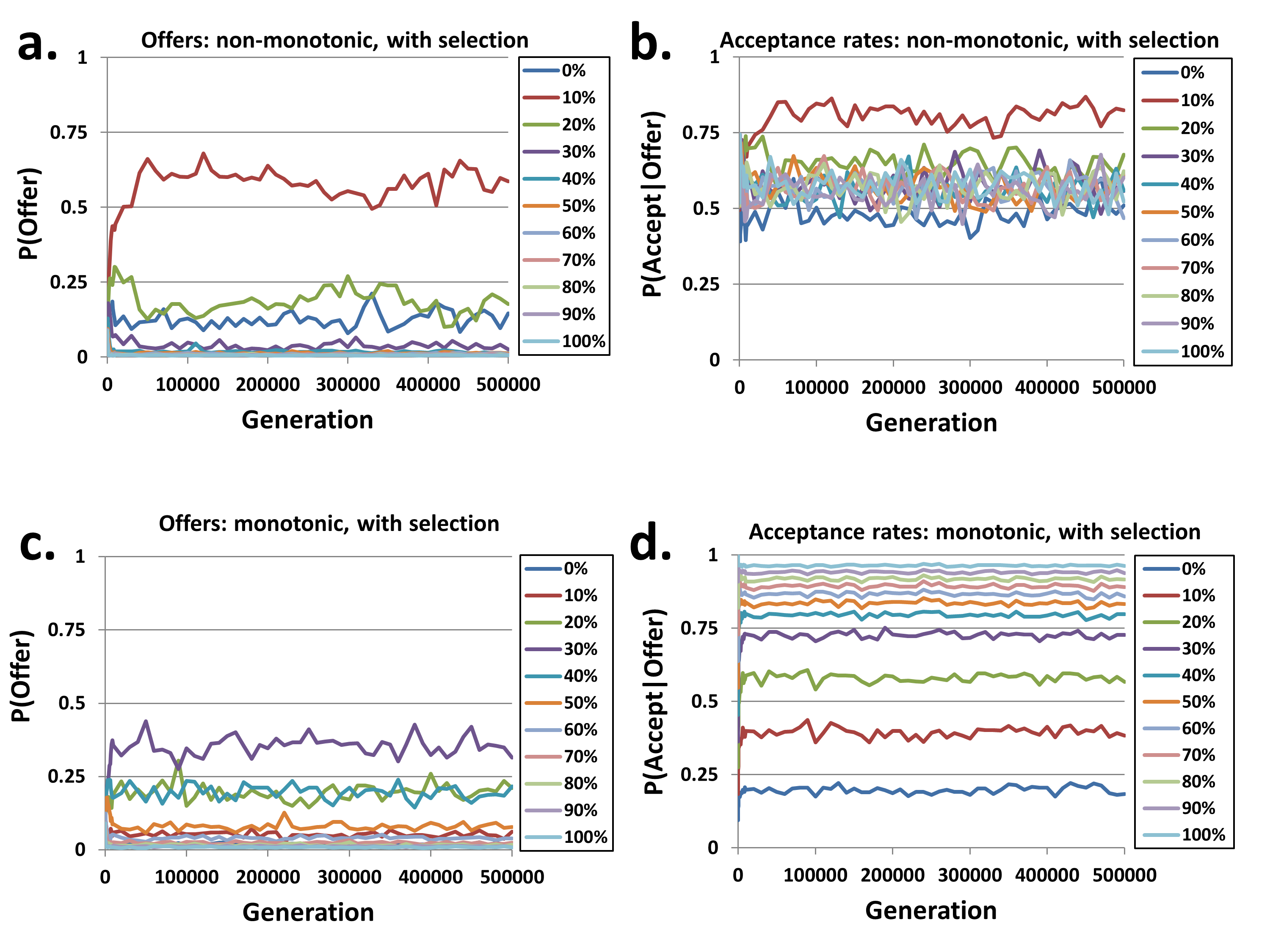

Supplement: S3 Fig — For all panels, each line represents the mean genotype across populations for each of the eleven offer sizes (N = 100 agents). Data are plotted across 500,000 generations of the simulation. As shown above, both non-monotonic and monotonic populations exhibited no systematic drift by the end of the simulations. (a) Mean genotypes for proposed offers in non-monotonic populations with selection pressure. (b) Mean genotypes for acceptance rates in non-monotonic populations with selection pressure. (c) Mean genotypes for proposed offers in monotonic populations with selection pressure. (d) Mean genotypes for acceptance rates in monotonic populations with selection pressure. (TIF) [file pone.0134636.s004.TIF]

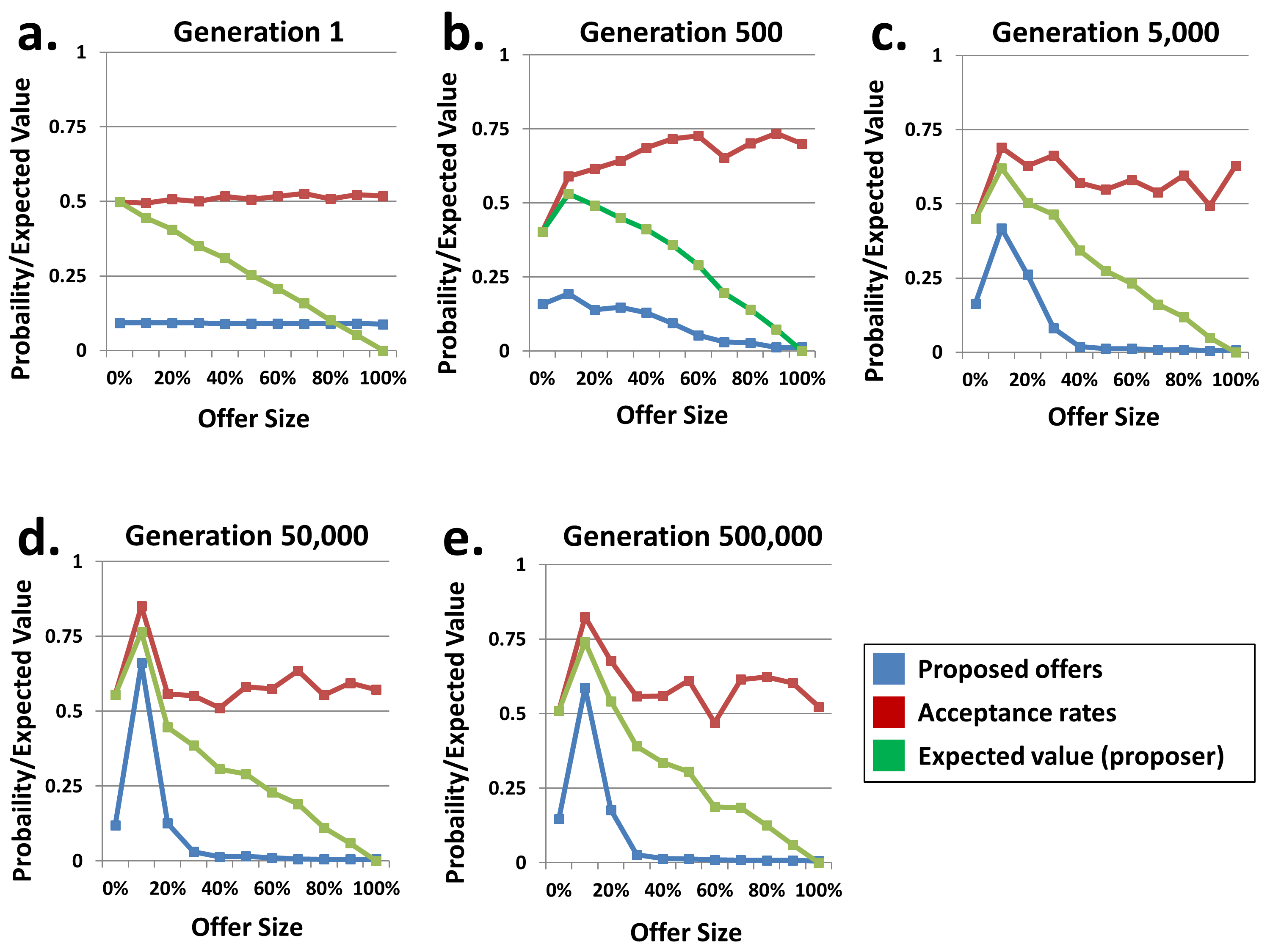

Supplement: S4 Fig — The sequence of panels depicts the evolutionary time course of proposer and responder genotypes for a single population size (N = 100 agents) when responders’ acceptance rates were not constrained to increase monotonically across offer sizes. The lines in each panel show the mean genotype for proposers (blue line) and responders (red line) across the eleven possible offers. The expected value of each offer, from the proposer’s perspective, is shown in green. When the population was initialized, neither mean offers nor acceptance rates differed significantly across the possible magnitudes and the highest expected value for proposers initially came from an offer of zero. However, the maximum expected value quickly shifted to the minimum non-zero offer (10%), as responders derived no fitness from accepting offers of zero, but did derive a small amount of fitness from low offers. As proposers and responders co-evolved, offers of 10% accounted for approximately half of all offers, with a negligible number of offers over 30% being made. (TIF) [file pone.0134636.s005.TIF]

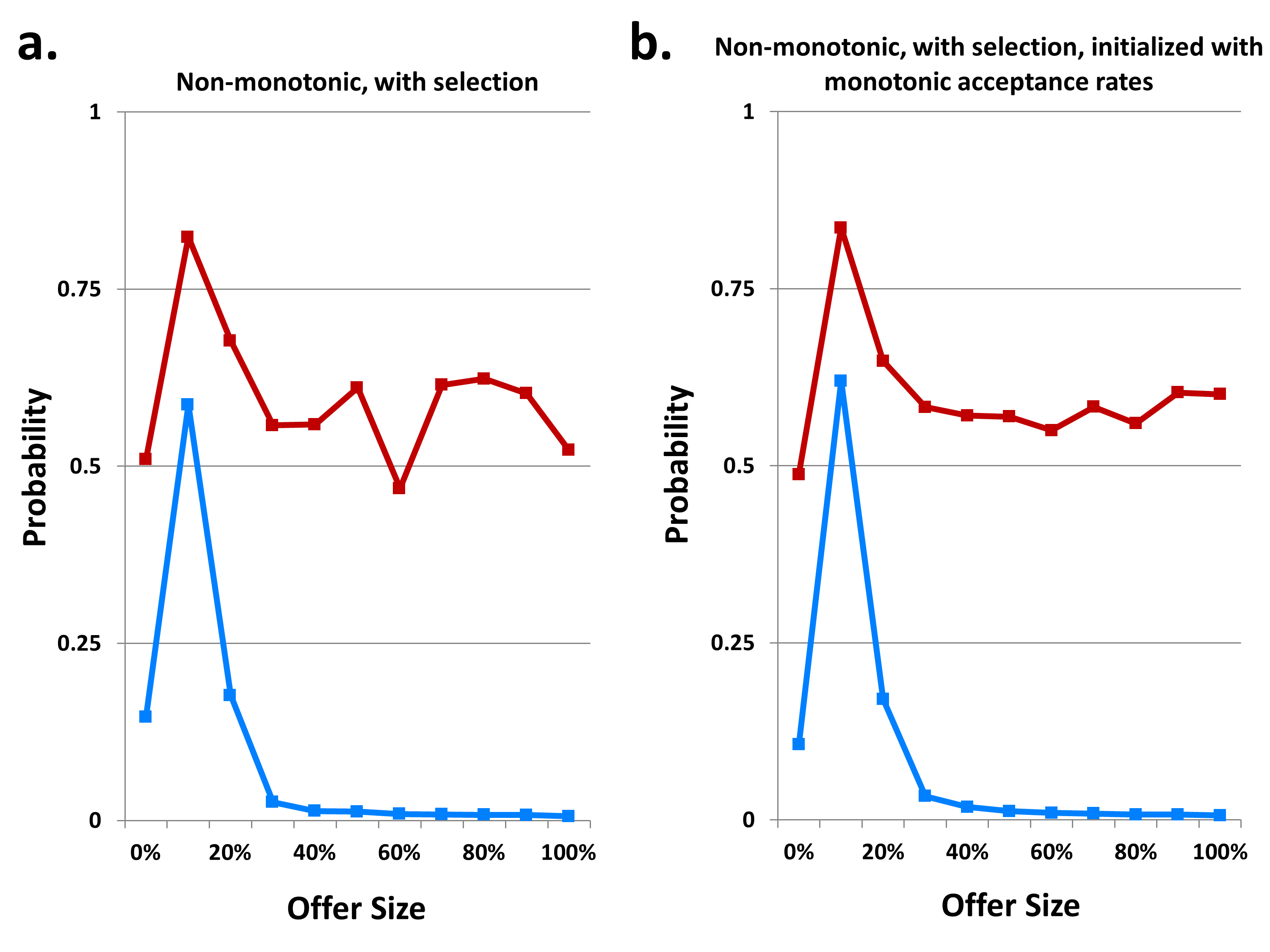

Supplement: S5 Fig — For each panel, mean genotypes of 1,000 populations (N = 100 agents) are shown for proposed offers (blue) and acceptance rates (red) after 500,000 generations. All responder genotypes were constrained to be monotonically increasing across offer sizes. (a) Data from the original, non-monotonic populations, with selection (Fig 1C and 1D), for purposes of comparison. (b) Data from simulations of non-monotonic populations that were initialized according to the procedure for monotonic populations. After initialization, populations evolved without the monotonic constraint on acceptance rates. After 500,000 generations, the resulting equilibria were not appreciably different: the most frequent offer was the lowest non-zero offer (i.e., 10% of the resource), and these low offers were frequently accepted. (TIF) [file pone.0134636.s006.TIF]

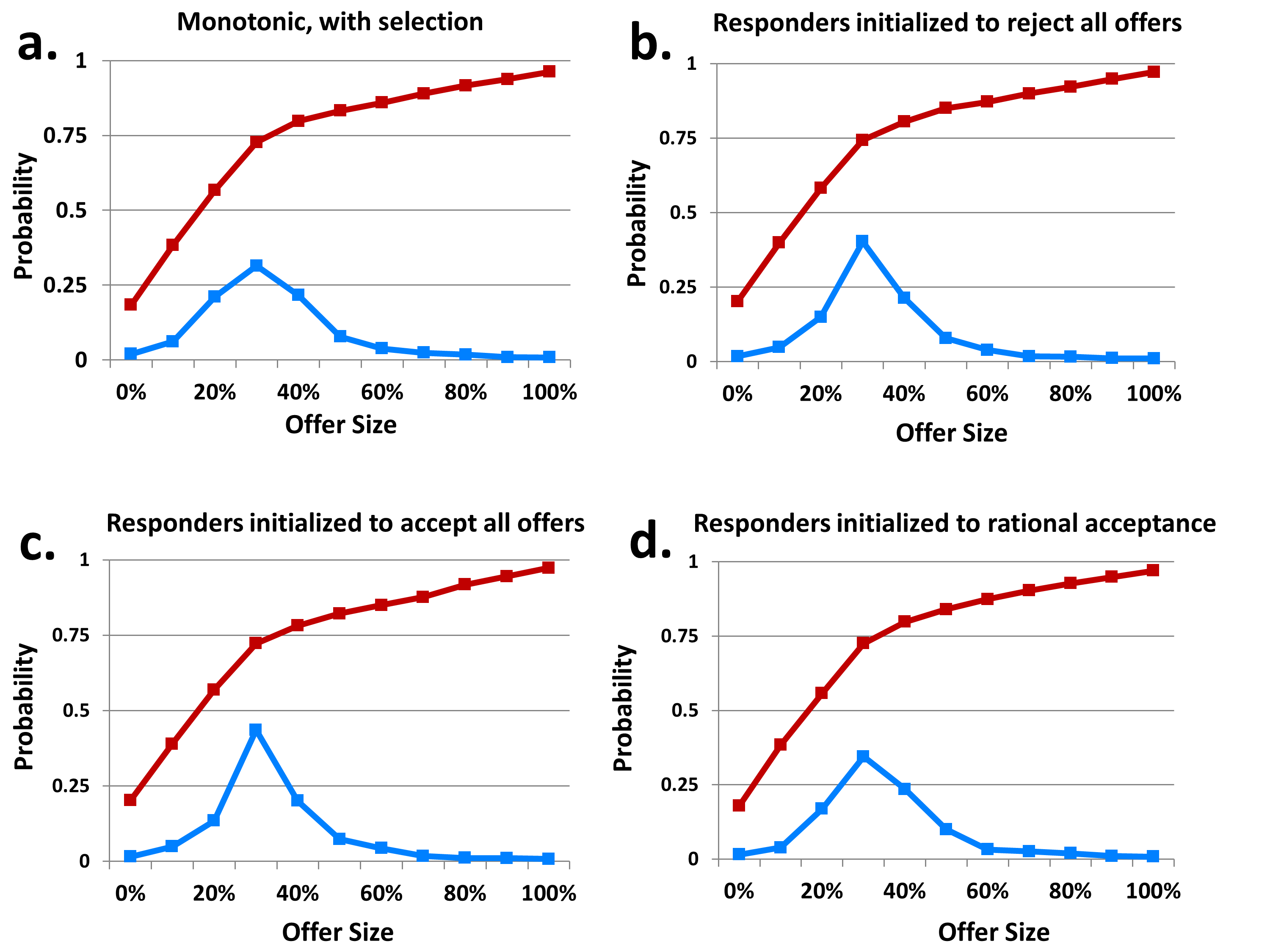

Supplement: S6 Fig — Here, a variety of alternative conditions are tested to examine the robustness of the evolution of fair offers for monotonic populations. For each panel, mean genotypes of 1,000 populations (N = 100 agents) are shown for proposed offers (blue) and acceptance rates (red) after 500,000 generations. Genotypes for acceptance rates were constrained to be monotonically increasing across offer sizes. As shown above, none of these alternate initializations appreciably changed the final equilibrium: proposers still evolved a modal offer of 30% and the functional forms of acceptance rates were similar to the original initialization. (a) Data from the original, monotonically constrained simulations, with selection pressure (Fig 2C and 2D). (b) Data from simulations in which responders initially rejected all offers (i.e., acceptance rates were initialized to zero for all loci). (c) Data from simulations in which responders initially accepted all offers (i.e., acceptance rates were initialized to one for all loci). (d) Data from simulations in which responders initially accepted all non-zero offers (i.e., acceptance rates were initialized to zero for the locus corresponding to 0%, and one for all other loci). (TIF) [file pone.0134636.s007.TIF]

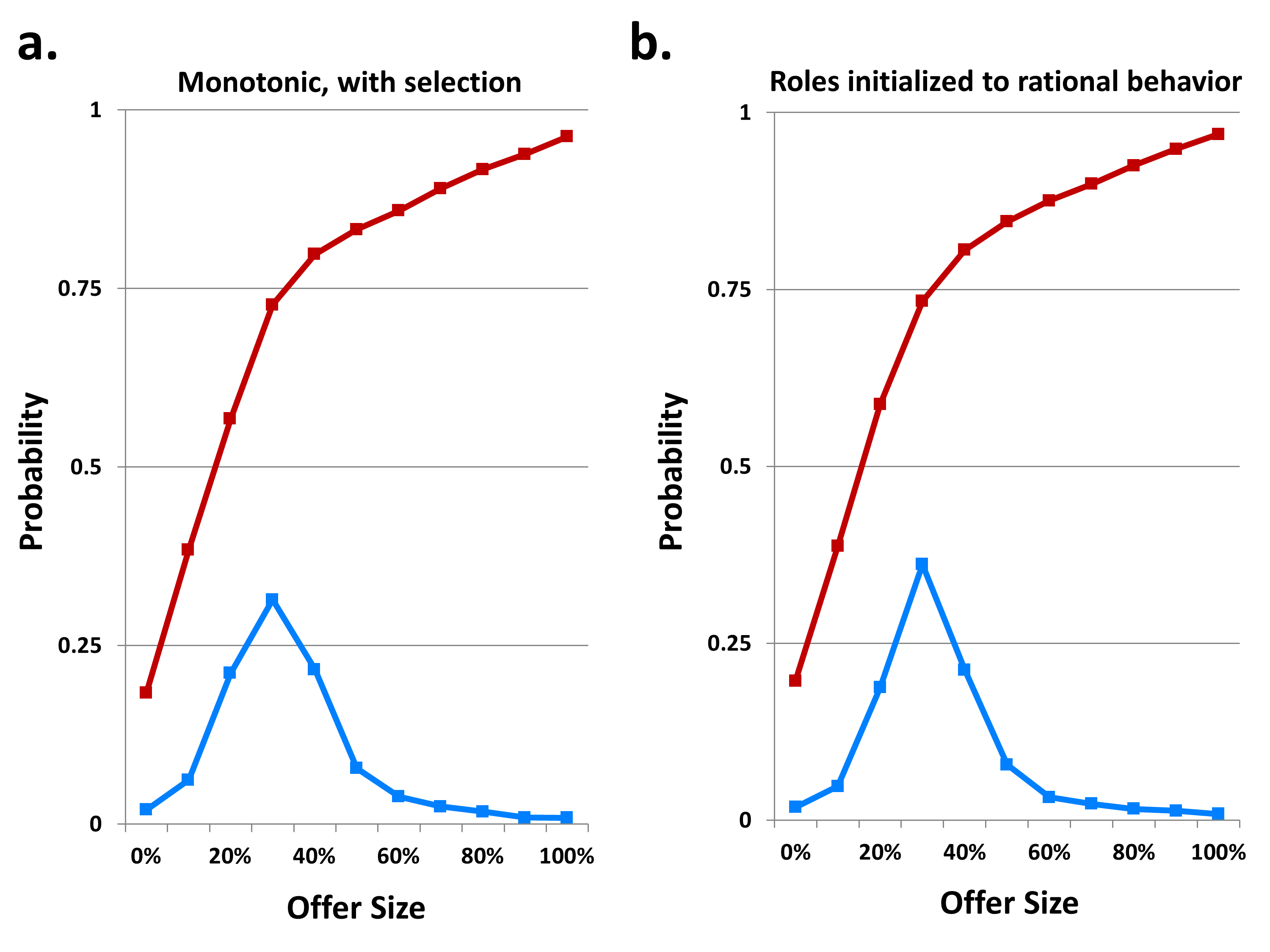

Supplement: S7 Fig — Here, an additional alternative condition is tested, in which both responder and proposer genotypes were initialized to the economically rational equilibrium. Responders were initialized to accept all non-zero offers (i.e., acceptance rates were initialized to zero for the locus corresponding to 0%, and one for all other loci) and proposed offers were initialized such that offers of 10% had a probability of one. For each panel, mean genotypes of 1,000 populations (N = 100 agents) are shown for proposed offers (blue) and acceptance rates (red) after 500,000 generations. Acceptance rates were constrained to be monotonically increasing across offer sizes. (a) Data from the original, monotonically constrained simulations, with selection pressure (Fig 2C and 2D). (b) Data from simulations with the economically rational initialization. This alternate initialization did not appreciably change the final equilibrium: proposers still evolved a modal offer of 30% and the functional forms of acceptance rates were similar to the original initialization. (TIF) [file pone.0134636.s008.TIF]

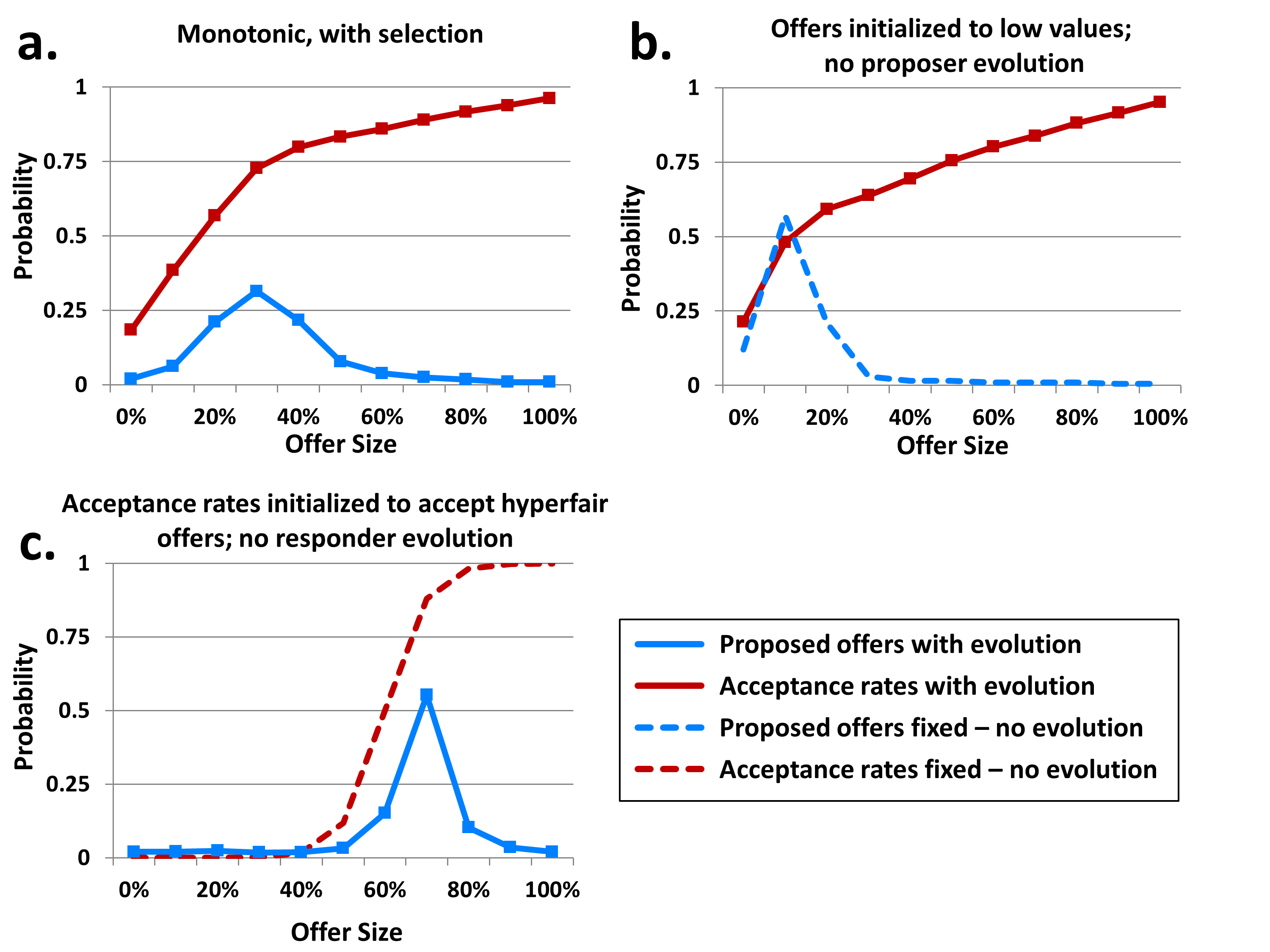

Supplement: S8 Fig — Here, alternative conditions are tested to examine the adaptability of population genotypes when only one role was allowed to evolve. For each panel, mean genotypes of 1,000 populations (N = 100 agents) are shown for proposed offers (blue) and acceptance rates (red) after 500,000 generations. Acceptance rates were constrained to be monotonically increasing across offer sizes. (a) Data from the original, monotonically constrained simulations (Fig 2C and 2D). (b) When proposers were constrained to make only low offers (with genotypes based on those from non-monotonic populations, Fig 1C) and did not evolve, acceptance rates for the minimum non-zero offer (10% of the resource) shifted from 38% to 48%. (c) When responders were initialized to accept only hyperfair offers and did not evolve, proposer genotypes evolved to make modal offers of 70%. Thus, each role could indeed adapt to the behavior of the other, and the genotypes exhibited by the original, monotonically constrained populations depended on the co-evolution of both roles. (TIF) [file pone.0134636.s009.TIF]

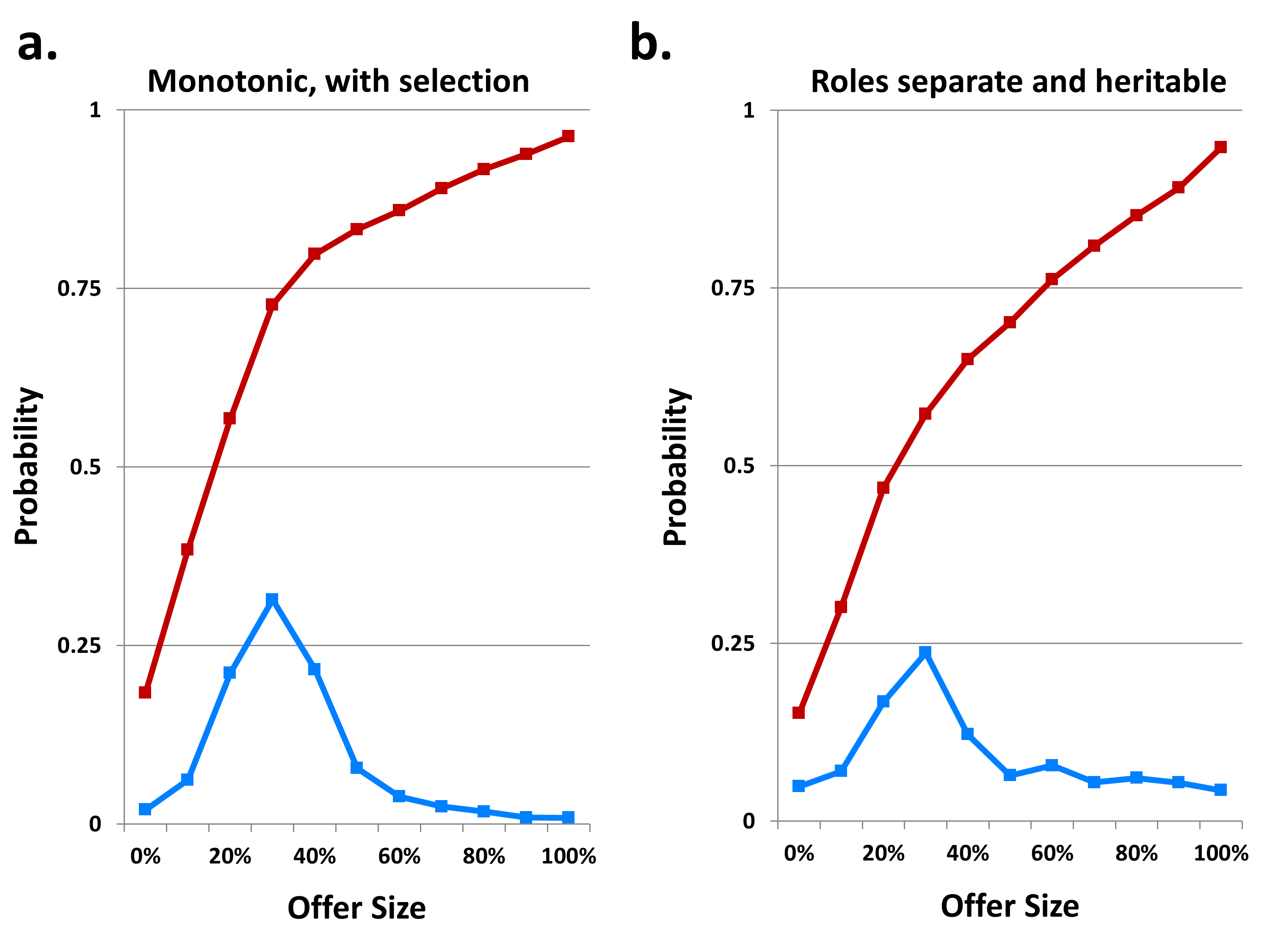

Supplement: S9 Fig — For each panel, mean genotypes of 1,000 populations (N = 100 agents) are shown for proposed offers (blue) and acceptance rates (red) after 500,000 generations. Acceptance rates were constrained to be monotonically increasing across offer sizes. (a) Data from the original, monotonically constrained simulations, with selection pressure (Fig 2C and 2D). (b) When proposers and responders were two separate but simultaneously evolving populations, results were similar (but not identical) to those in Fig 2C and 2D: the modal offer was 30%, and these offers were frequently accepted (although not as frequently as in the original simulation). That is, the norms co-evolved by the populations did not depend critically on the individuals’ ability to diversify the source of their fitness across the two roles. (TIF) [file pone.0134636.s010.TIF]

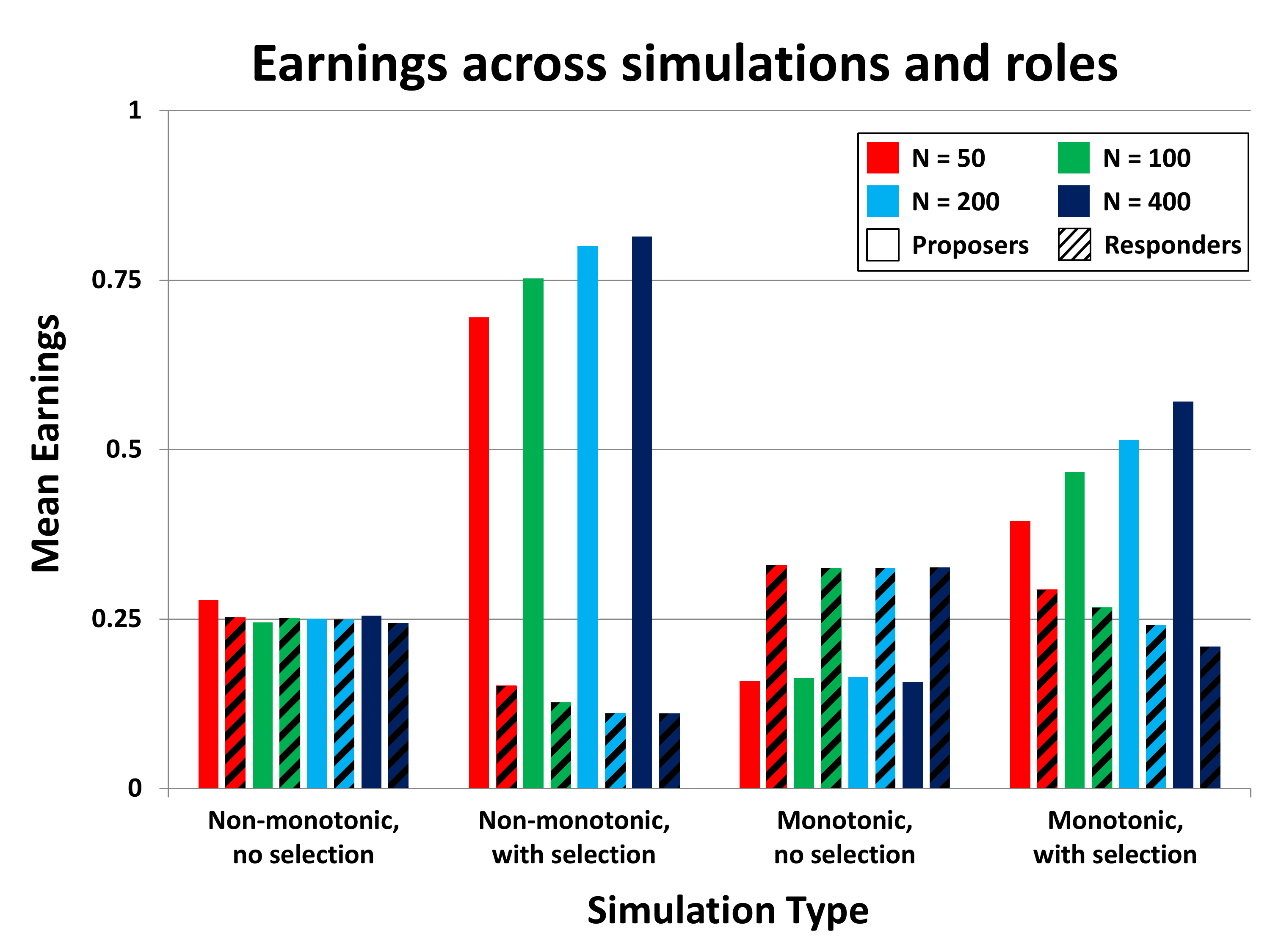

Supplement: S10 Fig — Mean earnings for proposers and responders are shown, grouped by the type of simulation. The earnings of agents that did not experience any form of selection pressure did not vary when acceptance rates were non-monotonic. For non-monotonic populations with selection (i.e., when acceptance rates were not monotonically constrained and reproduction depended upon earnings), proposers earned significantly more than responders, with the disparity accentuated for larger populations. This is not surprising, given the prevalence of low offers and their high rates of acceptance–a norm to which agents more strictly adhere as the population size increases. Earnings did not vary according to population size in the monotonically constrained populations without selection pressure. However, responders did earn more than proposers, as offers were uniformly distributed and acceptance rates for high offers were mathematically constrained to be higher than those for low offers. For monotonic populations with selection (i.e., when acceptance rates were monotonically constrained and reproduction depended upon earnings), the disparity in earnings between the roles was mitigated relative to non-monotonic populations with selection. (TIF) [file pone.0134636.s011.TIF]

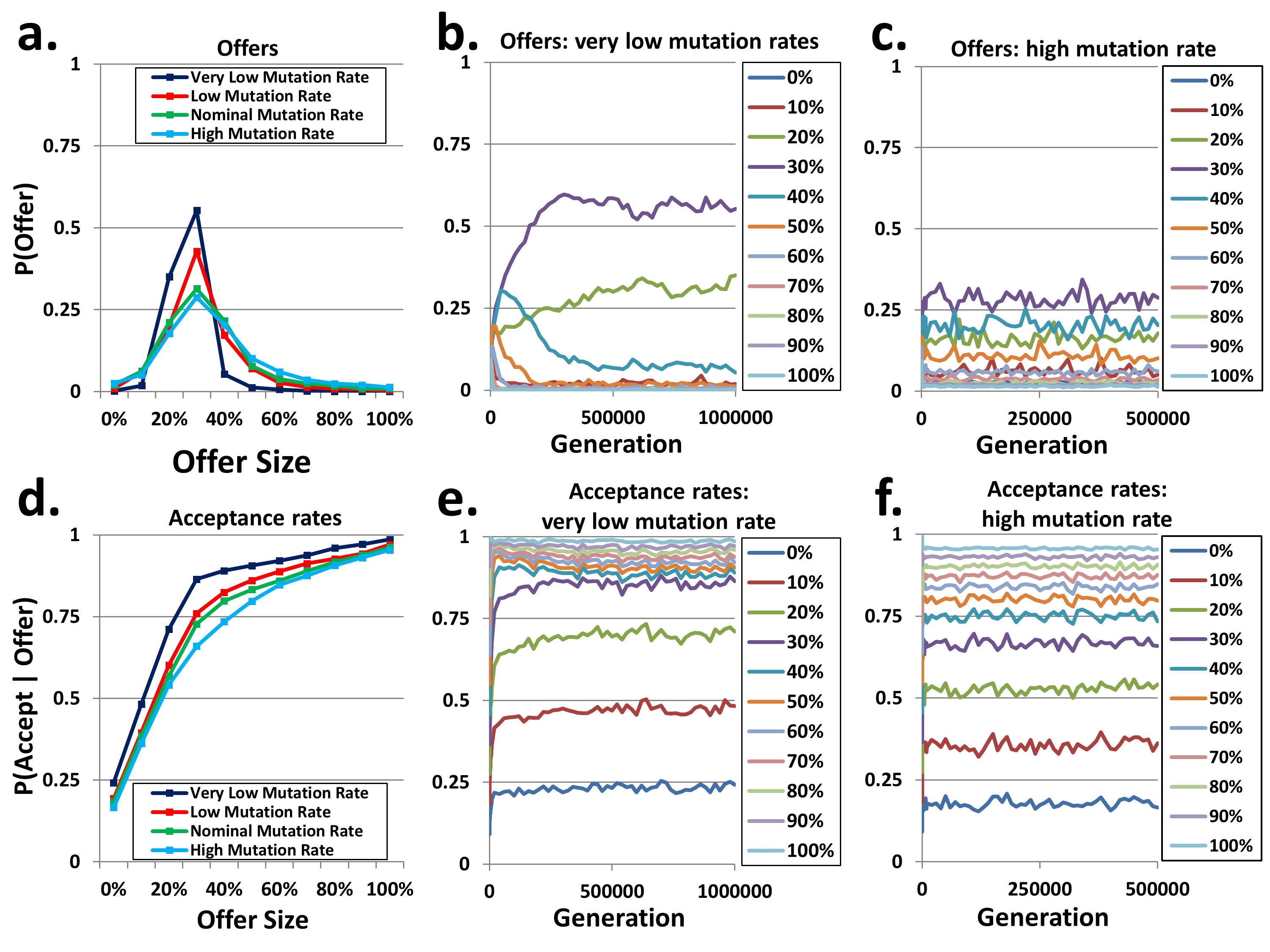

Supplement: S11 Fig — For panels a and d, mean genotypes of 1,000 populations (N = 100 agents) are shown for proposed offers (a) and acceptance rates (d) for multiple mutation rates. In addition to the nominal mutation rate used for all other simulations (a value of .2), mutation rates of .01, .1 and .4 (i.e., the inverse of the population size, half of the nominal rate, and double the nominal rate) were simulated with selection pressure. Acceptance rates were constrained to be monotonically increasing across offer sizes in all three simulations. For panels b, c, e and f, each line represents the mean genotype across populations for each of the eleven offer sizes (N = 100 agents). Data are plotted across 500,000 generations for the high mutation rate (panels c and f); in order to ensure that steady state was reached, simulations with the very low mutation rate were run for 1,000,000 generations and are plotted accordingly. (a) Mean offer probabilities under the four mutation rates. Modal offers remained at 30% regardless of the mutation rate possessed by the population. (b) Mean genotypes for proposed offers with a very low mutation rate. As shown above, offers exhibited no systematic drift by the end of the simulations. (c) Mean genotypes for proposed offers with a high mutation rate. As shown above, offers exhibited no systematic drift by the end of the simulations. (d) Mean acceptance rates under the four mutation rates. Although acceptance rates were slightly higher for lower mutation rates, the functional forms were qualitatively the same. (e) Mean genotypes for acceptance rates with a very low mutation rate. As shown above, acceptance rates exhibited no systematic drift by the end of the simulations. (f) Mean genotypes for acceptance rates with a high mutation rate. As shown above, acceptance rates exhibited no systematic drift by the end of the simulations. (TIF) [file pone.0134636.s012.TIF]
