# Supplementary material for: Rational Constraints and the Evolution of Fairness in the Ultimatum Game
Source: PLoS One. 2015 Jul 30;10(7):e0134636. doi: 10.1371/journal.pone.0134636 (PMC4520471; doi:10.1371/journal.pone.0134636)
Supplement: S12 Fig — Details regarding the screening of studies used to summarize human behavior in the Ultimatum Game. (PDF) [file pone.0134636.s013.pdf]

PRISMA 2009 Flow Diagram

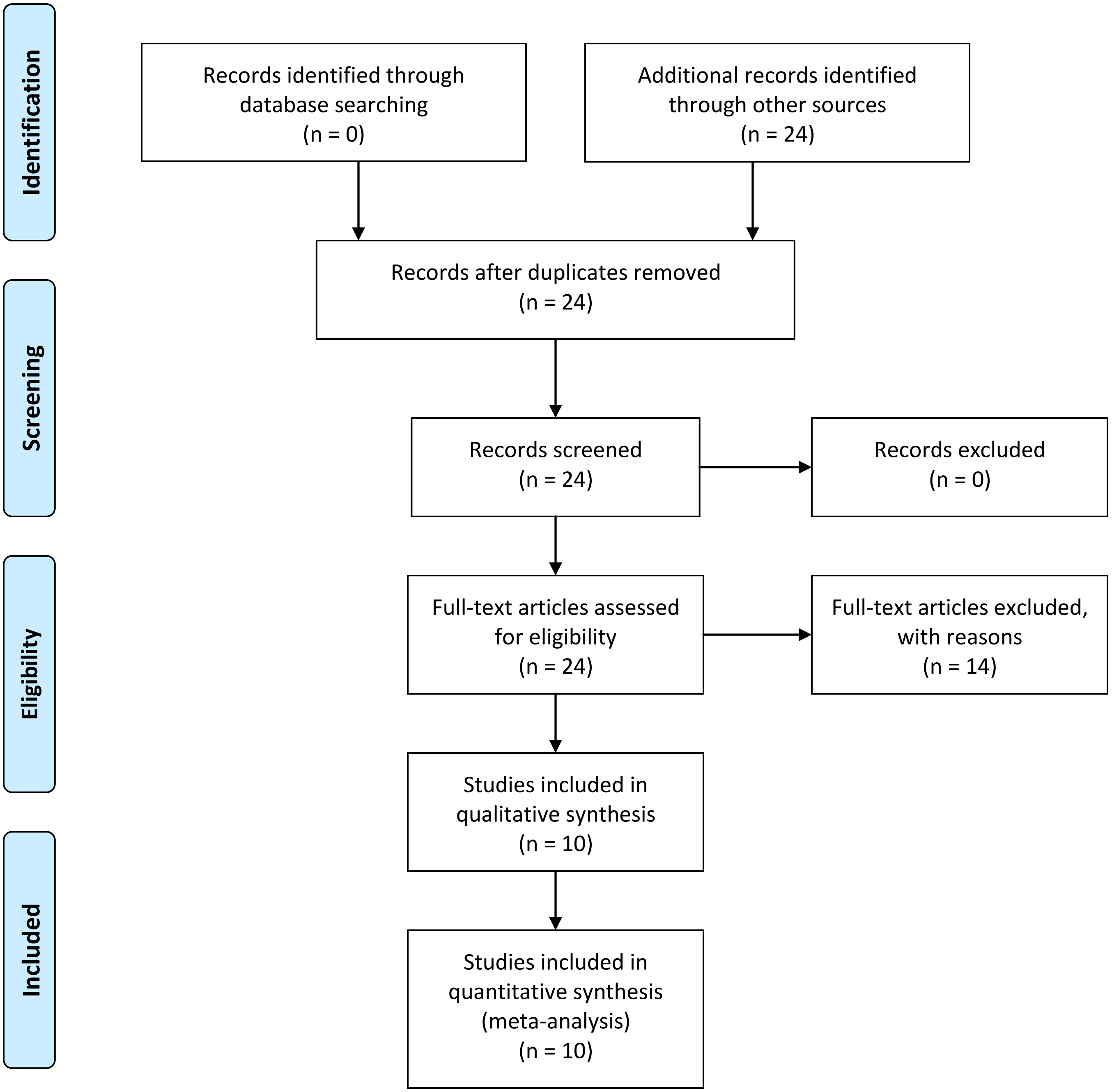

From: Moher D, Liberati A, Tetzlaff J, Altman DG, The PRISMA Group (2009). *P*referred *R*eporting *I*tems for *S*ystematic Reviews and *M*eta-*A*nalyses: The PRISMA Statement. PLoS Med 6(6): e1000097. doi:10.1371/journal.pmed1000097

For more information, visit www.prisma-statement.org.
